# Peroxisome-Deficiency and HIF-2α Signaling Are Negative Regulators of Ketohexokinase Expression

**DOI:** 10.3389/fcell.2020.00566

**Published:** 2020-07-08

**Authors:** Tanja Eberhart, Miriam J. Schönenberger, Katharina M. Walter, Khanichi N. Charles, Phyllis L. Faust, Werner J. Kovacs

**Affiliations:** ^1^Institute of Molecular Health Sciences, ETH Zürich, Zurich, Switzerland; ^2^Department of Biology, San Diego State University, San Diego, CA, United States; ^3^Department of Pathology and Cell Biology, Vagelos College of Physicians and Surgeons, Columbia University, New York, NY, United States

**Keywords:** EPAS1, HIF-2α, peroxisomes, ketohexokinase, VHL, fructose, Zellweger syndrome, Pex2

## Abstract

Ketohexokinase (KHK) is the first and rate-limiting enzyme of fructose metabolism. Expression of the two alternatively spliced KHK isoforms, KHK-A and KHK-C, is tissue-specific and KHK-C is predominantly expressed in liver, kidney and intestine and responsible for the fructose-catabolizing function. While KHK isoform choice has been linked to the development of disorders such as obesity, diabetes, cardiovascular disease and cancer, little is known about the regulation of total KHK expression. In the present study, we investigated how hypoxic signaling influences fructose metabolism in the liver. Hypoxia or von Hippel-Lindau (VHL) tumor suppressor loss leads to the stabilization of hypoxia-inducible factors alpha (HIF-1α and HIF-2α) and the activation of their signaling to mediate adaptive responses. By studying liver-specific *Vhl*, *Vhl*/*Hif1a*, and *Vhl*/*Epas1* knockout mice, we found that KHK expression is suppressed by HIF-2α (encoded by *Epas1*) but not by HIF-1α signaling on mRNA and protein levels. Reduced KHK levels were accompanied by downregulation of aldolase B (ALDOB) in the livers of *Vhl* and *Vhl/Hif1a* knockout mice, further indicating inhibited fructose metabolism. HIF-1α and HIF-2α have both overlapping and distinct target genes but are differentially regulated depending on the cell type and physiologic or pathologic conditions. HIF-2α activation augments peroxisome degradation in mammalian cells by pexophagy and thereby changes lipid composition reminiscent of peroxisomal disorders. We further demonstrated that fructose metabolism is negatively regulated by peroxisome-deficiency in a *Pex2* knockout Zellweger mouse model, which lacks functional peroxisomes and is characterized by widespread metabolic dysfunction. Repression of fructolytic genes in *Pex2* knockout mice appeared to be independent of PPARα signaling and nutritional status. Interestingly, our results demonstrate that both HIF-2α and peroxisome-deficiency result in downregulation of *Khk* independent of splicing as both isoforms, *Khka* as well as *Khkc*, are significantly downregulated. Hence, our study offers new and unexpected insights into the general regulation of KHK, and therefore fructolysis. We revealed a novel regulatory function of HIF-2α, suggesting that HIF-1α and HIF-2α have tissue-specific opposing roles in the regulation of *Khk* expression, isoform choice and fructolysis. In addition, we discovered a previously unknown function of peroxisomes in the regulation of fructose metabolism.

## Introduction

Dietary fructose is a monosaccharide naturally found in fruits and vegetables and is a major component of commonly used sweeteners like sucrose and high-fructose corn syrup (Jensen et al., 2018). In humans and rodents, fructose transport is mediated by solute carrier family 2 member 2 (SLC2A2) and SLC2A5, also known as GLUT2 and GLUT5, respectively. SLC2A5 has high specificity for fructose and is mainly expressed at the apical membrane of epithelial intestinal cells for fructose absorption, followed by renal proximal tubule cells for reabsorption (Douard and Ferraris, 2008). SLC2A2 facilitates the uptake of both glucose and fructose from the bloodstream into the cytoplasm. While SLC2A2 expression is highest in the liver, the primary organ metabolizing ingested fructose, basolateral membranes of epithelial intestinal and kidney cells as well as pancreatic β-cells also express considerable levels (Douard and Ferraris, 2013; Thorens, 2015). Ketohexokinase (KHK), or fructokinase, is the major fructose-metabolizing enzyme that catalyzes the conversion of fructose and ATP into fructose-1-phosphate (F1P) and ADP, respectively (Hayward and Bonthron, 1998). F1P is further metabolized by aldolase B (ALDOB) into dihydroxyacetone phosphate (DHAP) and glyceraldehyde that serve as substrates for the generation of glucose, glycogen and triglycerides (Geidl-Flueck and Gerber, 2017). Fructose phosphorylation by KHK occurs rapidly and without negative feedback regulation causing intracellular ATP depletion. There are two human inherited disorders where fructose metabolism is affected. Essential fructosuria (OMIM 229800) is a benign condition resulting from KHK deficiency. Affected individuals cannot phosphorylate fructose, so it rises to high levels in the serum and is excreted in the urine. In hereditary fructose intolerance (HFI; OMIM 229600), lack of ALDOB activity causes accumulation of F1P resulting in augmented phosphate depletion and subsequent uric acid generation after fructose intake. Blocking KHK activity has been demonstrated to reverse multiple deleterious manifestations of HFI such as hypoglycemia, hyperuricemia, hepatic inflammation or intestinal damage (Lanaspa et al., 2018). HFI patients must conform to a low-fructose diet and thus have an extremely low lifetime exposure to fructose.

While the existence of several alternatively spliced KHK isoforms has been described, only KHK-C and KHK-A that are generated via the specific excision of adjacent exons 3A and 3C, respectively, are translated into protein. Expression of these two isoforms is tissue-specific but generally mutually exclusive. Only KHK-C has a high binding affinity for fructose, whereas KHK-A has low fructose binding affinity and a high K_m_ for phosphorylation of fructose (∼7 mM). KHK-C is the pre-dominant isoform in the liver, kidney, and intestine and responsible for the fructose-catabolizing function (Hayward and Bonthron, 1998; Diggle et al., 2009). Several studies highlight the importance of KHK-C isoform expression in the progression of diabetes, liver disease or hypertension (Ishimoto et al., 2012; Doke et al., 2018; Lanaspa et al., 2018; Hayasaki et al., 2019). KHK-A is expressed in a wide range of other tissues at relatively low levels (Asipu et al., 2003; Diggle et al., 2009; Chabbert et al., 2019). Its substrates remained unknown and KHK-A has only recently been shown to act as protein kinase that directly phosphorylates phosphoribosyl pyrophosphate synthetase 1 (PRPS1) in the *de novo* nucleic acid synthesis pathway in human hepatocellular carcinoma cells (Li et al., 2016). Interestingly, an isoform switch from *Khka* to *Khkc* that is mediated by hypoxia-induced splicing has been shown to induce fructose metabolism in pathologic cardiac hypertrophy (Mirtschink et al., 2015).

Hypoxia-inducible factors (HIFs) are the master regulators of the adaptive response to low oxygen levels. HIFs form a heterodimer consisting of a stable ARNT/HIF-1β subunit and O_2_-sensitive HIF-α subunits (HIF-1α, HIF-2α/EPAS1). HIF-α subunits are constantly produced to respond quickly to changes in partial oxygen pressure. They are enzymatically hydroxylated on conserved proline residues by prolyl-hydroxylases (EGLN1-3), and targeted for proteasomal degradation by an ubiquitin ligase complex containing the von Hippel-Lindau (VHL) tumor suppressor protein under normoxic conditions. When O_2_ is scarce or when VHL is functionally lost, the HIF-α subunits are stabilized, dimerize with HIF-1β and together they interact with the transcriptional coactivators p300/Creb-binding protein. This transcriptional complex binds to hypoxia-response elements (HREs) in promoters of target genes and mediates a transcriptional response to hypoxia. Metabolic adaptations under low oxygen levels include enhanced glucose and glutamine uptake, glycolysis and glutaminolysis, and reduction of pyruvate catabolism by mitochondria and lipid synthesis (Nakazawa et al., 2016). Moreover, hypoxic signaling directly influences the abundance of high-oxygen consuming organelles such as mitochondria or peroxisomes specifically via HIF-1α or HIF-2α, respectively (Sowter et al., 2001; Zhang et al., 2007; Liu et al., 2012; Walter et al., 2014; Schönenberger and Kovacs, 2015).

Peroxisomes are subcellular single membrane-bound organelles with essential functions in a variety of metabolic processes such as the oxidation of very long-chain and branched-chain fatty acids, biosynthesis of bile acids, cholesterol, ether-linked phospholipids, and polyunsaturated fatty acids as well as metabolism of reactive oxygen species (Kovacs et al., 2002; Van Veldhoven, 2010; Fransen et al., 2012; Faust and Kovacs, 2014; Wanders et al., 2015). Peroxisomal function, number and size is cell type specific and highly dependent on metabolic demands. In mammals, peroxisomes are present in virtually every cell type except erythrocytes, with high abundance in liver and kidney (Islinger et al., 2018). Their physiological significance is highlighted by the existence of peroxisomal disorders in which either functional peroxisomes are absent (Zellweger Spectrum Disorders) or single enzyme deficiencies occur (Waterham et al., 2016). The severity of the manifestation and disease progression varies dramatically, depending on the peroxisomal defect. Characteristic diagnostic features of patients with peroxisomal disorders include accumulation of very long-chain fatty acids, bile acid intermediates, pristanic and phytanic acid, urinary oxalate and glycolate as well as reduced levels of plasmalogens and docosahexaenoic acid. These aberrations are associated with neuronal defects and developmental abnormalities, hepatomegaly and hepatic dysfunction or renal cyst formation and adrenal insufficiency (Waterham et al., 2016; Wanders, 2018). Moreover, aberrations in mitochondrial structure and functional defects in the electron transport chain at the inner mitochondrial membrane have been observed in liver biopsies from patients with peroxisome biogenesis disorders (PBD) (Goldfischer et al., 1973; Mooi et al., 1983; Trijbels et al., 1983; Hughes et al., 1990). Accordingly, distorted mitochondria as well as altered mitochondrial function have been described in peroxisome-deficient *Pex5* knockout mouse models with impaired gluconeogenesis, glycogen synthesis and insulin signaling but enhanced glycolysis in the liver (Baumgart et al., 2001; Peeters et al., 2011).

High fructose metabolism promotes the development of fatty liver (Lanaspa et al., 2012; Ishimoto et al., 2013), diabetes (Ishimoto et al., 2012; Lanaspa et al., 2014, 2018; Doke et al., 2018), and cancer (Ozawa et al., 2016; Goncalves et al., 2019). Moreover, myocardial hypoxia activates fructose metabolism in human and murine models of cardiac hypertrophy through HIF-1α-driven activation of splice factor 3b subunit 1 (*Sf3b1*) and SF3B1-mediated splice switching of KHK-A to KHK-C (Mirtschink et al., 2015). Additionally, heterogeneous nuclear ribonucleoprotein (HNRNP) H1 and H2 (Li et al., 2016) as well as APOBEC1 complementation factor (A1CF) have recently been shown to mediate KHK-A and KHK-C isoform expression via alternative splicing (Lin et al., 2018; Nikolaou et al., 2019). However, our general understanding about the regulation of fructolysis and the rate-limiting enzyme KHK is scarce. The liver is the central organ in carbohydrate metabolism, possesses high peroxisome abundance and is the organ with the highest KHK-C expression and enzyme activity (Diggle et al., 2009). Since HIF-2α signaling stimulates hepatic lipid accumulation and regulates peroxisome numbers (Rankin et al., 2009; Liu et al., 2014; Walter et al., 2014), organelles essential for metabolic homeostasis, we aimed to understand how hypoxic signaling and peroxisomes affect fructose metabolism in the liver.

## Materials and Methods

### Mice

*Albumin-Cre* [B6.Cg-Tg(Alb-cre)21Mgn/J], *Vhl*^*f/f*^ (C;129S-Vhl < tm1Jae > /J), *Hif1a*^*f/f*^ (B6.129-Hif1a < tm3Rsjo > /J), and *Epas1*^*f/f*^ (B6.Epas1 < tm1Mcs > /J) mice were purchased from the Jackson Laboratory. *Atg7*^*f/f*^ (B6.Cg-Atg7 < tm1Tchi >) and *Atg5*^*f/f*^ (B6.129S-Atg5 < tm1Myok >) mice were obtained from the RIKEN Bio Resource Center (Ibaraki, Japan; Komatsu et al., 2005; Hara et al., 2006). Liver-specific inactivation of *Vhl*, *Hif1a*, and *Epas1* was achieved by mating with *Albumin-Cre* mice. *Cre*-negative littermates were used as controls. *Vhl*, *Vhl/Hif1a*, and *Vhl/Epas1* mutant mice were in a mixed genetic background (BALB/c, 129Sv/J, and C57BL/6). Homozygous *Pex2*^–/–^ mice were obtained by breeding *Pex2* heterozygotes on a hybrid Swiss Webster-129 (SW/129) background (Faust et al., 2001). Mice had access to food and water *ad libitum* and were exposed to a 12:12-h light-dark cycle. For the purposes of this study, control mice consisted of either *Pex2*^+/+^ (wild-type) or *Pex2*^+/–^ genotypes (hereafter referred to as *Pex2*^+/^), as their biochemical characteristics were comparable to one another (Kovacs et al., 2004, 2009, 2012). Mice received a single daily gavage dose of 50 mg/kg body weight/day WY-14,643 (BML-GR200; Enzo Life Sciences) or the carrier methylcellulose (0.1%) (Walter et al., 2014). 3-methyladenine (3-MA) (2 mg/kg/day) was administered by intraperitoneal injection (Walter et al., 2014). All protocols for animal use and experiments were approved by the Veterinary Office of Zurich (Switzerland) and by the Institutional Animal Care and Use Committee of San Diego State University and Columbia University.

### Western Blot Analysis

Frozen liver and kidney tissue (1:10 w/v) was homogenized in RIPA buffer (20 mM Tris, pH 7.5; 150 mM NaCl; 1 mM EDTA; 1 mM EGTA; 1% NP-40; 1% sodium deoxycholate) containing protease and phosphatase inhibitors (cOmplete and PhosSTOP, respectively; Roche Diagnostics, Mannheim, Germany) using the Potter S homogenizer (Sartorius, Göttingen, Germany). Homogenates were incubated on ice for 30 min and centrifuged at 20000 *g* for 20 min at 4°C. Protein concentration was determined by the BCA method (#23225; Pierce, Rockford, IL, United States). Equal amounts of protein were subjected to SDS-polyacrylamide gel electrophoresis (SDS-PAGE) and transferred to Amersham Protran Supported 0.2 μM nitrocellulose (#10600015; GE Healthcare, Glattbrugg, Switzerland). After blocking for 1 h in TBST (Tris-buffered saline with 0.05% Tween 20) containing 1% bovine serum albumin (BSA), membranes were probed with the indicated antibodies overnight at 4°C. The membranes were incubated with horseradish peroxidase-conjugated secondary antibodies (Goat anti-guinea pig, #106-035-003; Jackson ImmunoResearch Laboratories; Goat anti-rabbit, #401393; Goat anti-mouse, #401253; Sigma-Aldrich) and developed using Clarity Western enhanced chemiluminescence substrate (#170-5060; BioRad). Membranes were exposed either to Super RX autoradiographic films (Fuji, Düsseldorf, Germany) or the Fusion Solo S imaging system. Antibodies are listed in Supplementary Table S1. Blots were semi-quantitatively analyzed by densitometry using ImageJ 1.52 v (National Institutes of Health).

### Subcellular Fractionation

Liver tissue was homogenized by one stroke of a Potter-Elvehjem homogenizer in 10 volumes (w/v) of homogenization buffer (20 mM Tris, pH 7.4; 2 mM MgCl_2_; 250 mM sucrose; 10 mM EDTA; 10 mM EGTA) containing protease inhibitors. The homogenate was centrifuged at 1,000 *g* for 5 min. The obtained pellet was rehomogenized in the same way and centrifuged at 1,000 *g* for 5 min. The pellet was rehomogenized and centrifuged at 1,000 *g* for 5 min to give the nuclear pellet. The post-nuclear supernatants were combined and centrifuged at 100,000 *g* for 30 min. The supernatant comprised the cytosolic fraction, and the pellet represented the membrane fraction. The nuclear pellet was resuspended in 150 μl of the nuclear lysis buffer (20 mM HEPES, pH 7.6; 25% (v/v) glycerol; 0.42 M NaCl; 1.5 mM MgCl_2_; 1 mM EDTA; 1 mM EGTA) containing protease inhibitors, rotated for 1 h at 4°C, and centrifuged at 18,000 *g* for 30 min. The supernatant comprised the nuclear fraction.

### RNA Isolation and Quantitative RT-PCR (qRT-PCR)

Total RNA was prepared from frozen tissues with RNeasy Mini Kit (QIAGEN, Hilden, Germany) and treated with DNase I. Quantitative RT-PCR (qRT-PCR) assays were performed as described previously (Kovacs et al., 2012). First-strand cDNA was synthesized with random hexamer primers using the High-Capacity RNA-to-cDNA Kit (No. 4368813; Applied Biosystems). qRT-PCR was performed on a Roche LightCycler LC480 instrument in duplicates using 10 ng cDNA, 7.5 pmol forward and reverse primers, and the 2x KAPA SYBR FAST qPCR Mastermix (No. KK4601; KAPA Biosystems). Thermal cycling was carried out with a 5 min denaturation step at 95°C, followed by 45 three-step cycles: 10 s at 95°C, 10 s at 60°C, and 10 s at 72°C. Melt curve analysis was carried out to confirm the specific amplification of a target gene and absence of primer dimers. Primer sequences are listed in Supplementary Table S2. Expression levels were calculated using the 2^–ΔΔCT^ method (Livak and Schmittgen, 2001). *18s* rRNA or *cyclophilin* (*Ppia*) were used as the invariant control.

### Adenovirus

Adenovirus expressing HIF-2α P405/531A [hereafter called Ad-HIF-2α(mt)] was generated as described previously (Walter et al., 2014). A Cre recombinase- and GFP-expressing adenovirus (Ad-Cre-GFP) was used to delete floxed sequences in *Atg5^*f/f*^/Vhl^*f/f*^* mice. Mice were injected with different amounts of plaque forming units (pfu) of adenovirus in 200 μl PBS into the tail vein. Mice were killed 6 days after adenovirus delivery. Virus expressing only GFP served as control (Ad-GFP). All Adenoviruses used were purchased from Viraquest (North Liberty, IA, United States).

### Statistical Analyses

Data are expressed as mean ± SD. When two groups where compared, statistical significance was evaluated by an unpaired, two-tailed Student’s *t*-test or an unpaired, two-tailed Student’s *t*-test with Welch’s correction when variances where significantly different. For multiple group analysis one-way ANOVA followed by Dunnett’s multiple comparisons test or two-way ANOVA followed by either Sidak’s or Tukey’s multiple comparisons test was performed. Data were assumed to be normally distributed. Homoscedasticity was assumed for two-way ANOVA. Statistical analyses were performed using GraphPad Prism version 8.2.0.

## Results

### HIF-α Signaling Decreases Hepatic *Khk* and *Aldob* Expression

**FIGURE 1:**
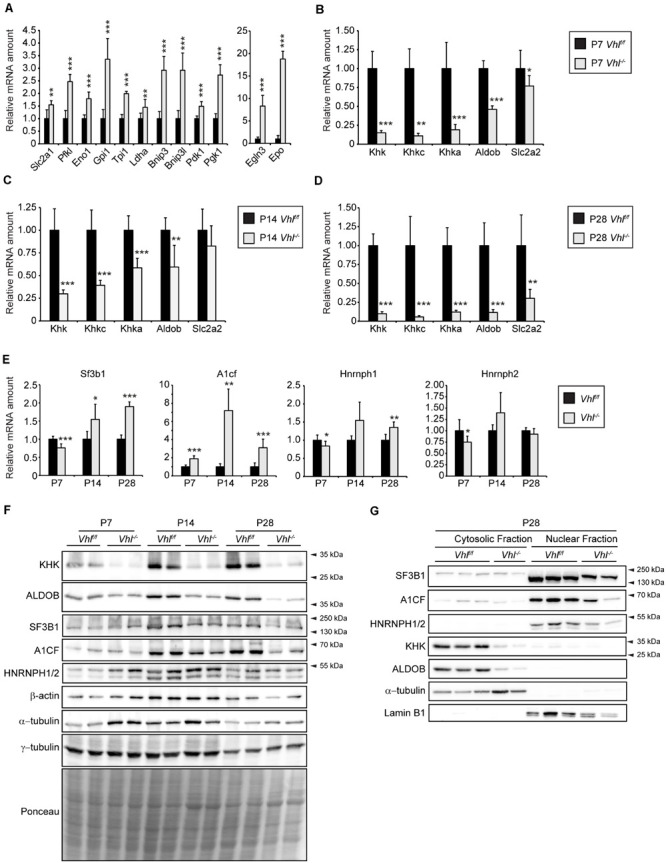
Analysis of the fructolytic pathway in *Vhl^–/–^* livers. The fructolytic pathway was analyzed in P7, P14, and P28 *Vhl*^*f/f*^ (control) and liver-specific *Vhl^–/–^* mice. **(A,B)** Expression of HIF-α target genes **(A)** and fructolytic genes **(B)** in P7 control and *Vhl^–/–^* livers. **(C)** Expression of fructolytic genes in P14 livers. **(D)** Expression of fructolytic genes in P28 livers. **(E)** Expression of splicing factors was analyzed in P7, P14, and P28 *Vhl*^*f/f*^ (control) and liver-specific *Vhl^–/–^* mice (*n* = 5–7 mice). **(F)** Immunoblots of liver lysates. **(G)** Immunoblots of cytosolic and nuclear fractions from livers of P28 mice with antibodies against SF3B1, A1CF, HNRNPH1/2, KHK, ALDOB, and α-tubulin or Lamin B1 as loading controls. Each value represents the amount of mRNA relative to that in control mice, which was arbitrarily defined as 1. *18S* rRNA and *cyclophilin* were used as the invariant control. Data are mean ± SD (*n* = 5–7 mice). Statistical analysis was performed using Student’s *t-*test or Student’s *t-*test with Welch’s correction. *p < 0.05; **p < 0.01; ***p < 0.001 vs. control mice.

To examine whether hypoxic signaling affects hepatic fructose metabolism or *Khk* isoform choice we first analyzed total *Khk* expression in liver-specific *Vhl* knockout mice (*Vhl^–/–^*) where both HIF-α isoforms are permanently stabilized, even under normoxic conditions. We previously described (Walter et al., 2014) that HIF-α target gene expression is induced in 2- and 4-week-old (P14 and P28, respectively) *Vhl^–/–^* mice. These mice were significantly smaller but had an increased liver to body weight ratio compared to control mice and developed hypoglycemia and severe steatosis. The hepatic expression of HIF-α target genes (*Slc2a1*, *Pfkl*, *Eno1*, *Gpi1*, *Tpi1*, *Pgk1*, *Ldha*, *Bnip3*, *Bnip3l*, *Pdk1*, *Egln3*, and *Epo*) was already induced in 1-week old (P7) *Vhl^–/–^* mice (Figure 1A). The expression of total *Khk* was significantly downregulated in P7, P14 and P28 *Vhl^–/–^* livers, which was reflected by reduced expression levels of *Khkc* as well as *Khka*. While *Aldob* was also transcriptionally downregulated at all time points analyzed, *Slc2a2* mRNA levels were reduced only in P28 *Vhl^–/–^* livers (Figures 1B–D). Concordantly, protein levels of total KHK and ALDOB were reduced in P7, P14, and P28 *Vhl^–/–^* livers compared to age-matched controls (Figure 1F). Recently, several studies showed that SF3B1 (Mirtschink et al., 2015), HNRNPH1/2 (Li et al., 2016) as well as A1CF mediate KHK-A and KHK-C isoform expression via alternative splicing (Lin et al., 2018; Nikolaou et al., 2019). Even though the strong decrease of *Khk* expression was reflected by reduced *Khkc* and *Khka* expression in the livers of *Vhl^–/–^* mice, we examined the expression of these *Khk* splicing mediators. *A1cf* mRNA expression was significantly upregulated in *Vhl^–/–^* livers, especially in P14 mice (∼7 fold). *Sf3b1* mRNA expression was increased ∼1.5- and ∼2-fold in P14 and P28 *Vhl^–/–^* mice, respectively, while levels of *Hnrnph1* and *2* where unchanged or only moderately altered in livers of *Vhl^–/–^* mice compared to their age-matched counterparts (Figure 1E). In contrast to the transcriptional upregulation, A1CF and SF3B1 protein levels were decreased in P14 and P28 whole liver lysates as well as in nuclear fractions of P28 *Vhl^–/–^* livers, while a moderate decrease of HNRNPH1/2 was observed (Figures 1F,G). As expected, KHK and ALDOB protein levels were strongly reduced in cytosolic fractions of P28 *Vhl^–/–^* livers (Figure 1G). Thus, these results indicate that HIF-α signaling impairs hepatic fructose catabolism via a mechanism that does not involve a KHK isoform switch.

### HIF-2α but Not HIF-1α Activation Represses *Khk* and *Aldob* Expression in *Vhl^–/–^* Livers

**FIGURE 2:**
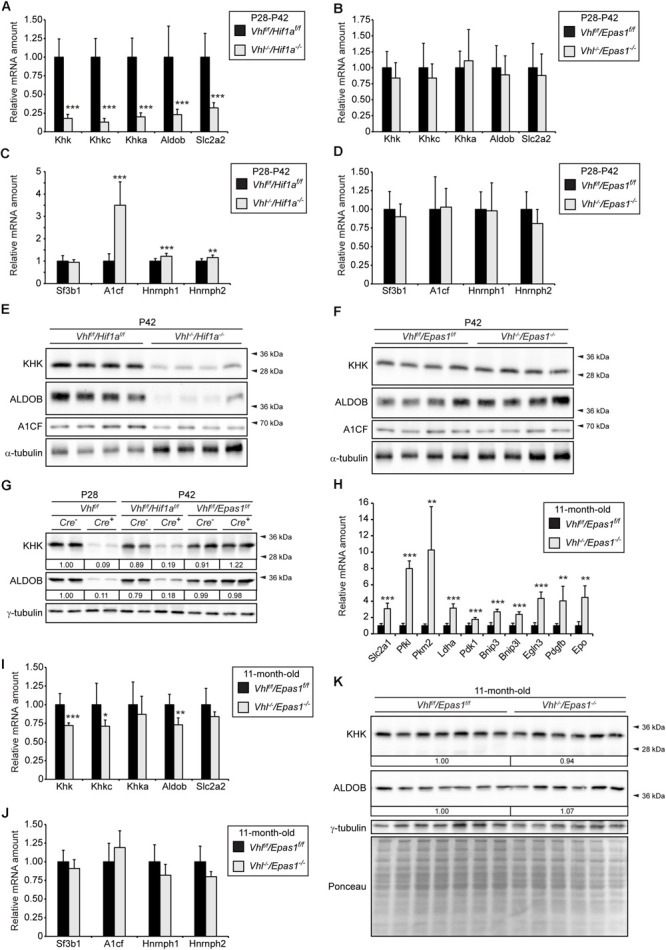
HIF-2α-dependent inhibition of fructolysis in *Vhl^–/–^* livers. The fructolytic pathway was analyzed in P28-P42 control (*Cre-*: *Vhl^*f/f*^/Hif1a^*f/f*^* and *Vhl^*f/f*^/Epas1^*f/f*^*) and liver-specific *Vhl^–/–^/Hif1a^–/–^* and *Vhl^–/–^/Epas1^–/–^* mice. **(A)** Expression of fructolytic genes in P28-P42 control and *Vhl^–/–^/Hif1a^–/–^* livers (*n* = 10 for P28-P42 mice; *n* = 4 for P28 mice; *n* = 6 for P42 mice). **(B)** Expression of fructolytic genes in P28-P42 control and *Vhl^–/–^/Epas1^–/–^* livers (*n* = 10 for P28-P42 mice; *n* = 4 for P28 mice; *n* = 6 for P42 mice). **(C)** Expression of splicing factors in P28-P42 control and *Vhl^–/–^/Hif1a^–/–^* livers (*n* = 10 mice). **(D)** Expression of splicing factors in P28-P42 control and *Vhl^–/–^/Epas1^–/–^* mice (*n* = 10 mice). **(E,F)** Immunoblots of liver lysates from P42 mice with antibodies against total KHK, ALDOB, A1CF, and α-tubulin as loading control. **(G)** Immunoblots of liver lysates from P28 or P42 mice with antibodies against total KHK, ALDOB, and γ-tubulin as loading control. Numbers at the bottom of the blots indicate the fold change as quantified after normalization with γ-tubulin. Protein levels were expressed relative to that in livers of *Vhl*^*f/f*^ mice, which were arbitrarily defined as 1. **(H**–**J)** Expression of HIF-α target genes **(H)**, fructolytic genes **(I)**, and splicing factors **(J)** in 11-month-old control and *Vhl^–/–^/Epas1^–/–^* livers (*n* = 8 for *Vhl^*f/f*^/Epas1^*f/f*^*, n = 6 for *Vhl^–/–^/Epas1^–/–^* mice). **(K)** Immunoblots of liver lysates from 11-month-old mice with antibodies against total KHK, ALDOB, and γ-tubulin as loading control. Numbers at the bottom of the blots indicate the fold change as quantified after normalization with γ-tubulin. Protein levels in *Vhl^–/–^/Epas1^–/–^* livers were expressed relative to that in control livers, which were arbitrarily defined as 1. In expression analyses, each value represents the amount of mRNA relative to that in control mice, which was arbitrarily defined as 1. The expression levels were similar in P28 and P42 mice and were therefore combined. *18S* rRNA and *cyclophilin* were used as the invariant control. Data are mean ± SD. Statistical analysis was performed using Student’s *t-*test or Student’s *t-*test with Welch’s correction. *p < 0.05; **p < 0.01; ***p < 0.001 vs. control mice.

To investigate whether the reduction of *Khk* and *Aldob* expression due to pVHL loss is mediated by HIF-1α or HIF-2α (encoded by *Epas1*), we determined the expression of fructolytic genes in liver-specific *Vhl/Hif1a* and *Vhl/Epas1* double knockout mice (Figure 2). The expression of total *Khk, Khkc, Khka*, *Aldob*, and *Slc2a2* was significantly reduced in P28-P42 *Vhl^–/–^/Hif1a^–/–^* mice (only HIF-2α signaling) compared to controls (Figure 2A). Changes in expression levels of fructolytic genes in *Vhl^–/–^/Hif1a^–/–^* and *Vhl^*f/f*^/Hif1a^*f/f*^* mice were similar to changes in *Vhl^–/–^* and *Vhl*^*f/f*^ mice (Supplementary Figure S1A). In contrast to *Vhl^–/–^* and *Vhl^–/–^/Hif1a^–/–^* mice, the mRNA levels of *Slc2a2* and fructolytic genes in P28-P42 *Vhl^–/–^/Epas1^–/–^* (only HIF-1α signaling) livers were not statistically different from those in *Vhl^*f/f*^/Epas1^*f/f*^* control livers, demonstrating a central role for HIF-2α in the suppression of fructolytic genes (Figure 2B and Supplementary Figure S1A). The increased expression of *A1cf* in *Vhl^–/–^* livers was mediated by HIF-2α, since *A1cf* expression was also increased in *Vhl^–/–^/Hif1a^–/–^* (Figure 2C) but remained unchanged in P28-P42 *Vhl^–/–^/Epas1^–/–^* livers compared to their respective controls (Figure 2D). In accordance with the gene expression data, total KHK and ALDOB protein levels were decreased in P42 *Vhl^–/–^/Hif1a^–/–^* but not in *Vhl^–/–^/Epas1^–/–^* livers compared to their controls (Figures 2E,F). A1CF protein levels were decreased in *Vhl^–/–^/Hif1a^–/–^* livers (Figure 2E), whereas A1CF levels were similar in control and *Vhl^–/–^/Epas1^–/–^* livers (Figure 2F). A direct comparison of KHK and ALDOB protein levels in livers of *Vhl^–/–^*, *Vhl^–/–^/Hif1a^–/–^*, and *Vhl^–/–^/Epas1^–/–^* with control mice confirmed that HIF-2α, but not HIF-1α, signaling suppresses fructolytic gene expression (Figure 2G and Supplementary Figure S1B). We previously showed that P28-P42 liver-specific *Vhl^–/–^/Epas1^–/–^* mice were phenotypically similar to control mice (Walter et al., 2014), with only a marginal increase in hepatic triacylglycerol levels in *Vhl^–/–^/Epas1^–/–^* livers. To examine if long-term HIF-1α signaling in the liver affects the expression of fructolytic genes, we analyzed 11-month-old liver-specific *Vhl^–/–^/Epas1^–/–^* mice. While the expression of HIF-1α target genes was significantly increased in *Vhl^–/–^/Epas1^–/–^* livers (Figure 2H), the expression of total *Khk*, *Khkc*, and *Aldob* was only marginally reduced over such a long period of HIF-1α signaling (Figure 2I). The expression of splicing factors was similar in 11-month-old control and *Vhl^–/–^/Epas1^–/–^* livers (Figure 2J). However, the protein levels of total KHK and ALDOB were comparable in 11-month-old control and *Vhl^–/–^/Epas1^–/–^* livers (Figure 2K).

**FIGURE 3:**
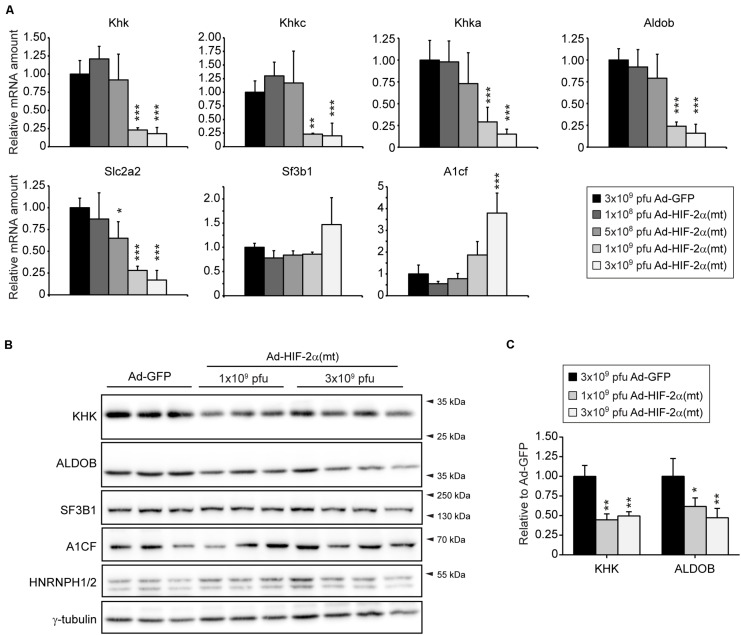
Overexpression of HIF-2α P405/531A [HIF-2α(mt)] inhibits the expression of fructolytic genes in the liver. **(A)** Expression of fructolytic genes and splicing factors in livers from 8-week-old *Atg7*^*f/f*^ mice infected with 3 × 10^9^ pfu of Ad-GFP (control) or different amounts of pfu of Ad-HIF-2α(mt) [Ad-GFP: *n* = 6; 3 × 10^9^ pfu Ad-HIF-2α(mt): *n* = 7; 1 × 10^9^, 5 × 10^8^, 1 × 10^8^ pfu Ad-HIF-2α(mt): *n* = 3–4]. Each value represents the amount of mRNA relative to that in GFP-infected mice. *Cyclophilin* was used as the invariant control. **(B)** Immunoblots of liver lysates with antibodies against total KHK, ALDOB, splicing factors (A1CF, HNRNPH1/2, and SF3B1) and γ-tubulin as loading control. **(C)** Quantification of KHK and ALDOB immunoblots after normalization with γ-tubulin. Data are mean ± SD. Statistical analysis was performed using one-way ANOVA followed by Dunnett’s multiple comparisons test. *p < 0.05; **p < 0.01; ***p < 0.001 vs. GFP-infected mice.

We used an additional approach to confirm that HIF-2α negatively affects the hepatic expression of fructolytic genes. Therefore, we examined mRNA and protein levels of KHK and ALDOB in livers from mice that had been injected with an adenovirus co-expressing GFP and HIF-2α P405/531A [hereafter called Ad-HIF-2α(mt)], a HIF-2α variant that escapes degradation by pVHL, or an adenovirus expressing GFP (Ad-GFP) as control. Mice were sacrificed 6 days after injection (Walter et al., 2014). Low doses of Ad-HIF-2α(mt) (1 × 10^8^ or 5 × 10^8^ pfus) showed little, if any, induction of HIF target genes, whereas the expression of HIF target genes was strongly increased in livers from mice infected with 1 × 10^9^ or 3 × 10^9^ pfu of Ad-HIF-2α(mt) (Walter et al., 2014). Infection with 1 × 10^9^ or 3 × 10^9^ pfu of Ad-HIF-2α(mt) also led to loss of peroxisomes and steatosis (Walter et al., 2014). The repression of fructolytic genes was dependent on virus quantity and mRNA levels of total *Khk*, *Khkc*, *Khka*, *Aldob*, and *Slc2a2* were strongly downregulated in mice infected with 1 × 10^9^ or 3 × 10^9^ pfu of Ad-HIF-2α(mt) (Figure 3A). *A1cf* expression was increased in livers from mice infected with 3 × 10^9^ pfu of Ad-HIF-2α(mt) to a similar extent as observed in livers of *Vhl^–/–^/Hif1a^–/–^* compared to correspondent controls (Figure 3A). In accordance with the gene expression data, protein levels of KHK and ALDOB were reduced in livers infected with 1 × 10^9^ or 3 × 10^9^ pfu of Ad-HIF-2α(mt) compared to controls (Figures 3B,C). The reduction in hepatic KHK and ALDOB protein levels after Ad-HIF-2α(mt) expression was less prominent (∼50% reduction for KHK and ALDOB) than observed in livers of *Vhl^–/–^* and *Vhl^–/–^/Hif1a^–/–^* mice suggesting considerable stability of the proteins. A1CF, SF3B1, and HNRNPH1/2 protein levels were unchanged in HIF-2α(mt)-expressing livers (Figure 3B). Taken together, we provide strong evidence that HIF-2α represses hepatic fructose metabolism. In addition, the splicing factors A1CF, SF3B1, and HNRNPH1/2 are not relevant for the direct regulation of *Khk* in the described models.

### *Khk* mRNA and Protein Levels Are Decreased in Peroxisome-Deficient Livers and Kidneys

**FIGURE 4:**
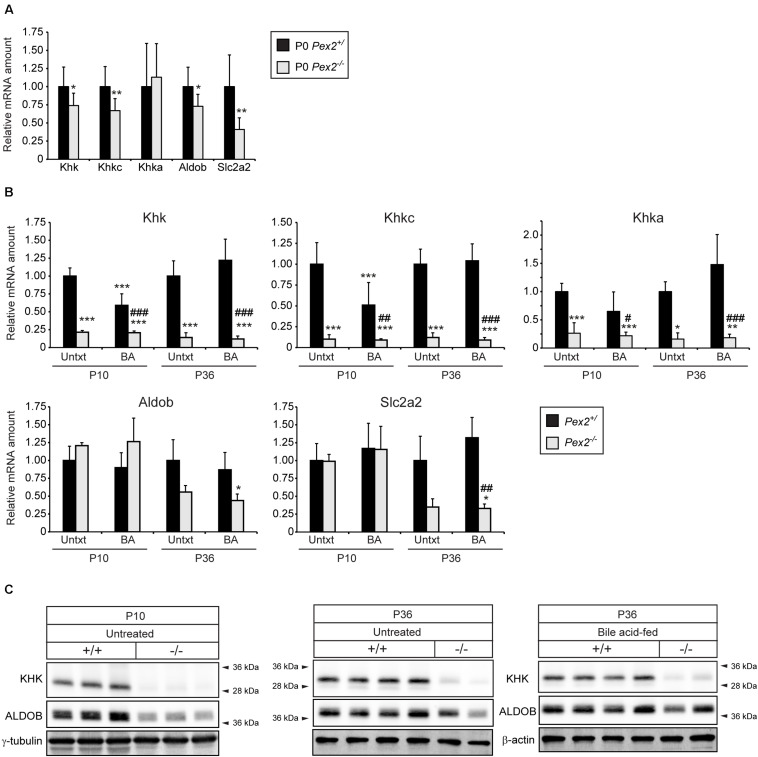
Analysis of the fructolytic pathway in livers from untreated and BA-fed control and *Pex2^–/–^* mice. **(A)** P0 liver (*n* = 9 for control and *Pex2^–/–^* mice). **(B)** P10 and P36 liver (*n* = 6 for untreated P10 control and *Pex2^–/–^* mice, *n* = 5 for P10 BA-fed control and *Pex2^–/–^* mice, *n* = 7 for untreated P36 control mice, *n* = 2 for untreated P36 *Pex2^–/–^* mice, *n* = 3 for P36 BA-fed control mice, *n* = 4 for P36 BA-fed *Pex2^–/–^* mice). **(C)** Immunoblots of liver lysates with antibodies against total KHK, ALDOB, and β-actin or γ-tubulin as loading controls. Each value represents the amount of mRNA relative to that in untreated control mice at that age, which was arbitrarily defined as 1. *18S* rRNA or cyclophilin were used as the invariant control. Data are mean ± SD. Statistical analysis was performed using Student’s *t-*test or Student’s *t-*test with Welch’s correction or two-way ANOVA followed by Sidak’s multiple comparisons test. *p < 0.05; **p < 0.01; ***p < 0.001 vs. untreated P0 or P10 or P36 control mice. ^#^*p* < 0.05; ^##^*p* < 0.01; ^###^*p* < 0.001 vs. P10 or P36 BA-fed control mice.

**FIGURE 5:**
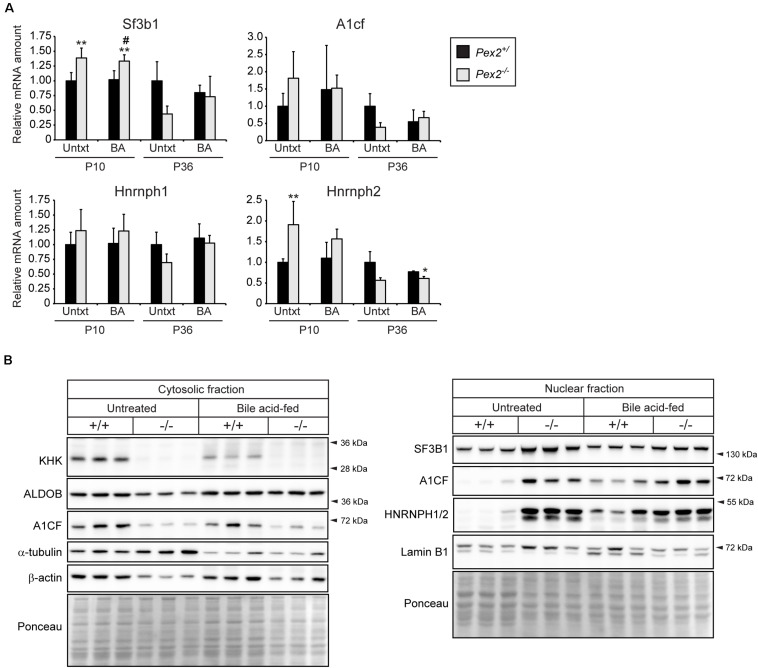
Analysis of splicing factors in livers from untreated and BA-fed control and *Pex2^–/–^* mice. **(A)** Expression of splicing factors in P10 and P36 control and *Pex2^–/–^* livers (*n* = 6 for P10 control and *Pex2^–/–^* mice, *n* = 5 for P10 BA-fed control and *Pex2^–/–^* mice, *n* = 7 for P36 control mice, *n* = 2 for P36 *Pex2^–/–^* mice, *n* = 3 for P36 BA-fed control mice, *n* = 4 for P36 BA-fed *Pex2^–/–^* mice). **(B)** Immunoblots of cytosolic and nuclear fractions from livers of untreated and BA-fed P10 control and *Pex2^–/–^* mice with antibodies against SF3B1, A1CF, HNRNPH1/2, and β-actin, α-tubulin, and Lamin B1 as loading controls. Each value represents the amount of mRNA relative to that in untreated control mice at that age, which was arbitrarily defined as 1. *18S* rRNA or cyclophilin were used as the invariant control. Data are mean ± SD. Statistical analysis was performed using two-way ANOVA followed by Sidak’s multiple comparisons test. *p < 0.05; **p < 0.01 vs. untreated control mice. ^#^*p* < 0.05 vs. BA-fed control mice.

HIF-2α is a major driver of lipid accumulation in the liver and triggers the degradation of peroxisomes via selective autophagy (Walter et al., 2014). Therefore, we investigated whether peroxisome abundance has an influence on genes and proteins involved in fructolysis. We analyzed livers of peroxisome-deficient *Pex2^–/–^* mice on a Swiss Webster × 129S6/SvEv (SW/129) genetic background that survive 1–3 weeks (rarely 5 weeks) (Faust et al., 2001). *Pex2^–/–^* mice are characterized by severe growth retardation, intestinal malabsorption, hepatic cholestasis and steatosis as well as dysregulated cholesterol homeostasis and ER stress (Kovacs et al., 2004, 2009, 2012). Feeding *Pex2^–/–^* mice with bile acids (BA) improves intestinal lipid absorption, reduces cholestasis and accumulation of toxic bile acid intermediates and thus prolongs survival (Keane et al., 2007). Total *Khk, Khkc, Aldob*, and *Slc2a2* expression levels were reduced by ∼25–50% in livers of newborn (P0) *Pex2^–/–^* mice (Figure 4A). Strikingly, the expression of total *Khk* as well as the isoforms *Khkc* and *Khka* was strongly reduced in livers of untreated and BA-fed P10 and P36 *Pex2^–/–^* mice, while *Aldob* and *Slc2a2* expression was reduced only in P36 knockout livers. BA treatment reduced the expression of total *Khk* and *Khkc* in P10 control mice (Figure 4B). Total KHK protein levels were barely detectable in livers of P10 and P36 untreated and BA-fed *Pex2^–/–^* mice (Figures 4C, 5B). In contrast to the transcriptional expression, ALDOB protein levels were decreased in P10 but normalized in P36 *Pex2^–/–^* livers compared to controls (Figure 4C). *Sf3b1*, *A1cf*, and *Hnrnph2* expression was moderately increased in untreated and BA-fed P10 *Pex2^–/–^* livers, whereas no significant changes were observed in P36 livers. Expression of *Hnrnph1* was similar in control and *Pex2^–/–^* livers (Figure 5A). Interestingly, A1CF protein levels were decreased in cytosolic fractions and increased in nuclear fractions of untreated and BA-fed P10 *Pex2^–/–^* livers, indicating that peroxisome-deficiency causes a shift in the subcellular localization of A1CF (Figure 5B). In addition, SF3B1 and HNRNPH1/2 protein levels were increased in nuclear fractions of livers from both untreated and BA-fed *Pex2^–/–^* mice (Figure 5B). Taken together, these results illustrate that fructose metabolism is repressed in the absence of functional peroxisomes, and the repression is not affected by improving the nutritional state of *Pex2^–/–^* mice with BA feeding. The repression of fructose metabolism is independent of a *Khk* splicing change, since *Khk*, *Khkc*, and *Khka* mRNAs and total KHK protein expression were decreased in *Pex2^–/–^* livers.

**FIGURE 6:**
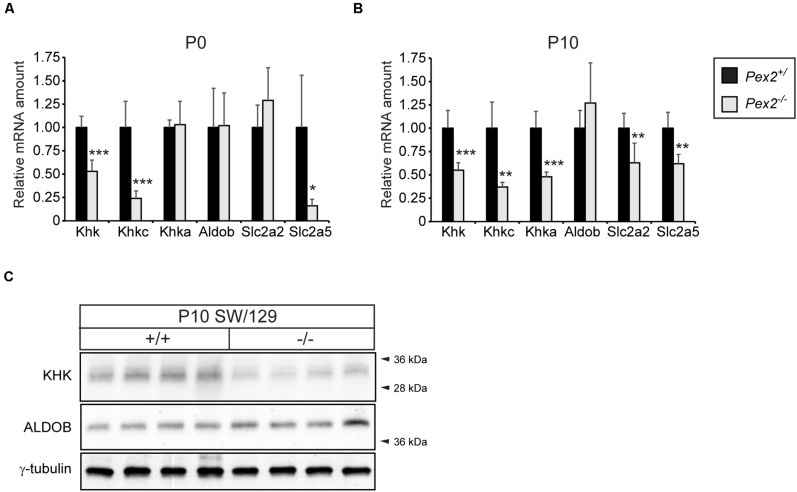
Analysis of the fructolytic pathway in kidneys from control and *Pex2^–/–^* mice. **(A)** Expression of fructolytic genes in P0 control and *Pex2^–/–^* kidneys (*n* = 6 for control, *n* = 4 for *Pex2^–/–^* mice). **(B)** Expression of fructolytic genes in P10 control and *Pex2^–/–^* kidneys (*n* = 6 for control and *Pex2^–/–^* mice). **(C)** Immunoblots of P10 kidney lysates with antibodies against total KHK, ALDOB, and γ-tubulin as loading control. Each value represents the amount of mRNA relative to that in control mice, which was arbitrarily defined as 1. *Cyclophilin* was used as the invariant control. Data are mean ± SD. Statistical analysis was performed using Student’s *t-*test or Student’s *t-*test with Welch’s correction. *p < 0.05; **p < 0.01; ***p < 0.001 vs. control mice.

Since the fructose metabolizing KHK-C isoform is also highly expressed in the proximal tubules of the kidney, where peroxisomes are highly abundant, we investigated the expression of fructolytic genes in kidneys of *Pex2^–/–^* mice. Similar to what we observed in the liver, total *Khk* as well as *Khkc* expression was significantly downregulated in P0 and P10 *Pex2^–/–^* kidneys while *Khka* mRNA levels were only reduced in P10 mice. The expression of *Aldob* was similar in P0 or P10 control and *Pex2^–/–^* kidneys. While *Slc2a2* expression was significantly decreased in P10 *Pex2^–/–^* kidneys, *Slc2a5* mRNA levels were reduced in both P0 and P10 *Pex2^–/–^* kidneys (Figures 6A,B). In agreement with the mRNA expression data, total KHK protein levels were reduced in P10 *Pex2^–/–^* kidneys while ALDOB protein levels were similar to controls (Figure 6C). In summary, these results confirm that fructose metabolism, especially KHK mRNA and protein expression, is reduced in the absence of functional peroxisomes in tissues with a generally high fructolytic activity.

### Repression of *Khk* Expression Is Independent of PPARα Signaling

**FIGURE 7:**
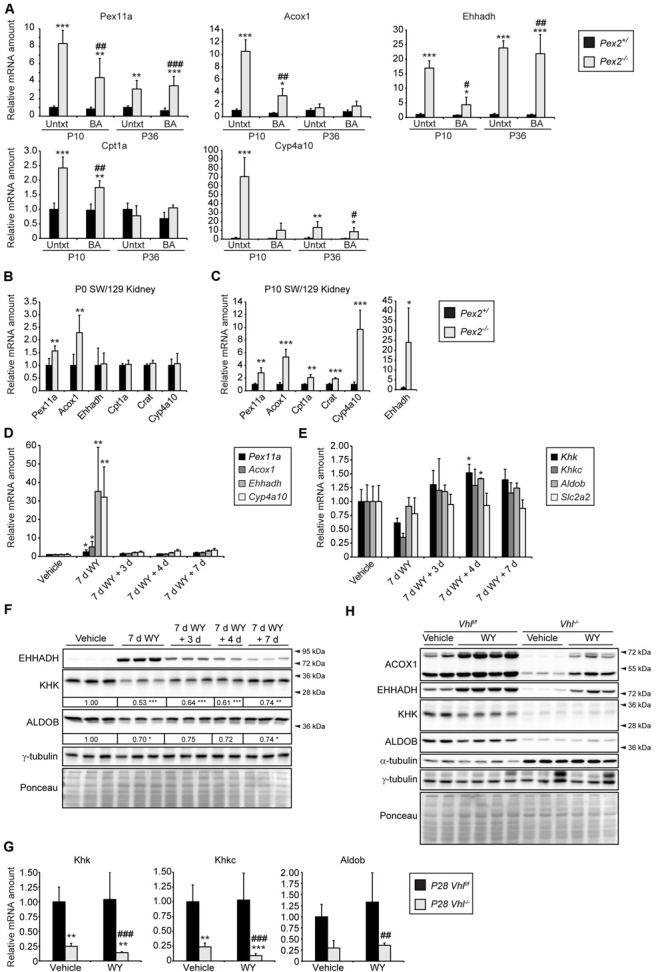
PPARα activation has only a minor effect on fructolysis. **(A)** Expression of PPARα target genes in P10 and P36 livers (*n* = 6 for P10 control and *Pex2^–/–^* mice, *n* = 5 for P10 BA-fed control and *Pex2^–/–^* mice, *n* = 7 for P36 control mice, *n* = 2 for P36 *Pex2^–/–^* mice, *n* = 3 for P36 BA-fed control mice, *n* = 4 for P36 BA-fed *Pex2^–/–^* mice). **(B,C)** Expression of PPARα target genes in P0 and P10 control and *Pex2^–/–^* kidneys (*n* = 6 for P0 control, *n* = 4 for P0 *Pex2^–/–^* mice; *n* = 6 for P10 control and *Pex2^–/–^* mice). **(D,E)** Expression of PPARα target **(D)** and fructolytic genes **(E)** in livers of adult C57BL/6J mice after treatment with the PPARα agonist WY-14,643 (WY) for 7 days (7 days) and after 3, 4, and 7 days after WY withdrawal (*n* = 3 for each experimental group). **(F,H)** Immunoblots of liver lysates with antibodies against ACOX1, EHHADH, KHK, ALDOB, and α-tubulin or γ-tubulin as loading controls. The fold change in the protein level in WY-treated C57BL/6J mice was expressed relative to that in vehicle-treated mice, which was arbitrarily defined as 1. **(G)** Expression of fructolytic genes in livers of vehicle- or WY-treated *Vhl*^*f/f*^ and liver-specific *Vhl^–/–^* mice (Vehicle: *n* = 4–8 mice; WY: *n* = 4–8 mice). Three-week-old mice have been treated with WY for 7 days. Each value represents the amount of mRNA relative to that in control mice, which was arbitrarily defined as 1. *Cyclophilin* was used as the invariant control. Data are mean ± SD. Statistical analysis was performed using Student’s *t-*test or Student’s *t-*test with Welch’s correction. For multiple group analysis one-way ANOVA or two-way ANOVA followed by Dunnett’s or by Sidak’s multiple comparisons test, respectively, was performed. *p < 0.05; **p < 0.01; ***p < 0.001 vs. control or vehicle-treated mice. ^#^*p* < 0.05; ^##^*p* < 0.01; ^###^*p* < 0.001 vs. P10 or P36 BA-fed control mice or WY-treated *Vhl*^*f/f*^ mice.

Next, we examined if the decreased *Khk* expression in *Pex2^–/–^* mice is mediated by peroxisome proliferator-activated receptor α (PPARα), a nuclear receptor that acts as a sensor for fatty acids and fatty acid derivatives and thus controls metabolic pathways involved in lipid and energy homeostasis (Pyper et al., 2010). Endogenous PPARα ligands such as CoA thioesters of very long-chain and branched-chain fatty acids are metabolized in peroxisomes (Hostetler et al., 2005, 2006). We have shown that the hepatic expression of PPARα target genes was either unchanged or significantly decreased in P0 *Pex2^–/–^* mice (Kovacs et al., 2012). In addition, PPARα target genes were not activated in the liver of *Acox1^–/–^* mice during the embryonic period, but their expression was induced as early as 1 day postnatal (Cook et al., 2001). These data suggest that the accumulation of abnormal metabolites may not yet be sufficient to induce the PPARα pathway in P0 *Pex2^–/–^* and *Acox1^–/–^* mice. However, accumulation of diet-derived unmetabolized substrates in peroxisome-deficient tissues in the postnatal period hyperactivates PPARα, as demonstrated by upregulation of PPARα target genes (*Pex11a*, *Acox1*, *Ehhadh*, *Cpt1a*, *Cyp4a10*) in the liver of P10 and P36 *Pex2^–/–^* mice (Figure 7A). Whereas only *Pex11a* and *Acox1* mRNA levels were induced in P0 *Pex2^–/–^* kidneys (Figure 7B), the expression of several PPARα target genes was increased in P10 *Pex2^–/–^* kidneys (Figure 7C). In summary, PPARα could mediate repression of fructolytic genes at least in postnatal *Pex2^–/–^* livers and kidneys.

In rodents, peroxisome proliferation is induced via pharmacological activation of PPARα (Schrader et al., 2012). To examine whether pharmacological activation of PPARα influences the expression of fructolytic genes, we analyzed livers from adult C57BL/6J mice after treatment with the PPARα agonist WY-14,643 (WY) for 7 days as well as after 3, 4 and 7 days after WY withdrawal. PPARα target genes *Pex11*α, *Acox1*, *Ehhadh*, and *Cyp4a10* were highly induced after the treatment with WY, and the mRNA levels returned almost to basal levels 3, 4, and 7 days after WY withdrawal (Figure 7D). Expression levels of total *Khk* and *Khkc* were reduced after 7 days of WY treatment and tended to be somewhat elevated after WY withdrawal, whereas WY treatment did not affect *Aldob* and *Slc2a2* mRNA expression (Figure 7E). WY treatment for 7 days strongly increased EHHADH protein levels and resulted in reduced KHK and ALDOB protein levels, which returned to levels observed in untreated mice over time after WY withdrawal (Figure 7F).

Next, we analyzed the effect of PPARα activation on the expression of fructolytic genes in livers of 4-week-old *Vhl^–/–^* mice after WY treatment for 7 days. WY treatment for 7 days strongly increased peroxisomal protein levels and catalase activity in control livers and to a lesser extent in *Vhl^–/–^* livers (Walter et al., 2014). We showed that the expression of PPARα target genes was significantly increased in both control and *Vhl^–/–^* mice upon WY treatment. However, WY-mediated induction of PPARα target genes was much weaker in *Vhl^–/–^* mice (Walter et al., 2014), suggesting that HIF-2α signaling impairs ligand-induced PPARα activation. This observation was confirmed by increased protein levels of ACOX1 and EHHADH after WY treatment in both wild-type and, to a lesser extent, in *Vhl^–/–^* mice compared to untreated controls (Figure 7H). However, expression of total *Khk*, *Khkc* as well as *Aldob* was not affected by WY treatment in control and *Vhl^–/–^* mice (Figure 7G). WY treatment slightly decreased KHK and ALDOB protein levels in control mice, but no further decrease could be observed in *Vhl^–/–^* livers (Figure 7H). Taken together, these results suggest that pharmacological PPARα activation exhibits at most moderate effects on KHK and ALDOB mRNA and protein levels, which also appear to depend on age, while HIF-2α signaling and peroxisome deficiency are strong repressors of fructolytic gene expression.

**FIGURE 8:**
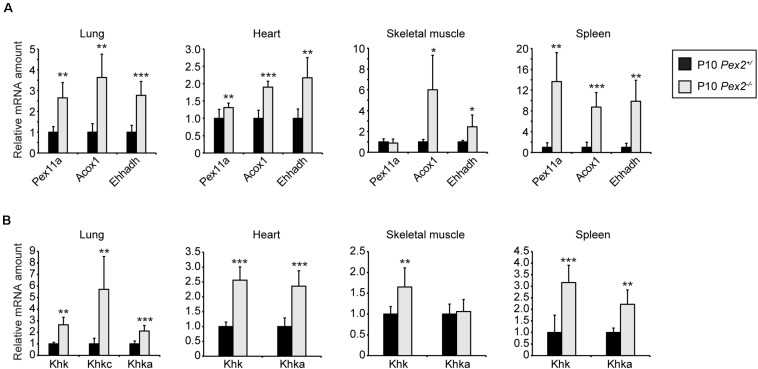
Expression of fructolytic genes in lung, heart, skeletal muscle, and spleen from control and *Pex2^–/–^* mice. **(A,B)** Expression of PPARα target **(A)** and fructolytic genes **(B)** in P10 control and *Pex2^–/–^* tissues (*n* = 6 for control and *Pex2^–/–^* mice). Each value represents the amount of mRNA relative to that in control mice, which was arbitrarily defined as 1. *Cyclophilin* was used as the invariant control. Data are mean ± SD (*n* = 6 for control and *Pex2^–/–^* mice). Statistical analysis was performed using Student’s *t-*test or Student’s *t-*test with Welch’s correction. *p < 0.05; **p < 0.01; ***p < 0.001 vs. control mice.

As KHK-A is the predominant isoform in extrahepatic tissues with relatively low fructose catabolic activity, we analyzed *Khk* expression in lung, heart, skeletal muscle, and spleen of P10 control and *Pex2^–/–^* mice. As expected, PPARα was also activated in these *Pex2^–/–^* tissues as indicated by transcriptional upregulation of the PPARα target genes *Pex11a*, *Acox1*, and *Ehhadh* (Figure 8A). Surprisingly, total *Khk* as well as *Khka* expression was increased in lung, heart, and spleen while only total *Khk* was more highly expressed in skeletal muscle in *Pex2^–/–^* mice compared to controls. The expression levels of *Aldob* were very low in these tissues and could not be analyzed. *Khkc* expression was only detectable in the lung at relatively low levels (C_T_ ∼37 in *Pex2*^+/^) and its mRNA level was higher in *Pex2^–/–^* mice, similar to the total *Khk* and *Khka* expression (Figure 8B). Total KHK protein levels were not detectable in lung, heart, skeletal muscle and spleen, probably due to the low *Khk* expression levels in these tissues.

In summary, loss of peroxisomes affects fructose metabolism in a tissue-specific manner that is dependent on KHK isoform dominance. There is a strong decrease of KHK mRNA and protein levels in postnatal *Pex2^–/–^* livers and kidneys, whereas *Khk* mRNA levels are increased in lung, heart, skeletal muscle, and spleen. Finally, these expression changes occur independent of PPARα activation.

### Decreased Peroxisome Abundance Is Not Required for HIF-2α-Mediated Inhibition of *Khk* Expression

**FIGURE 9:**
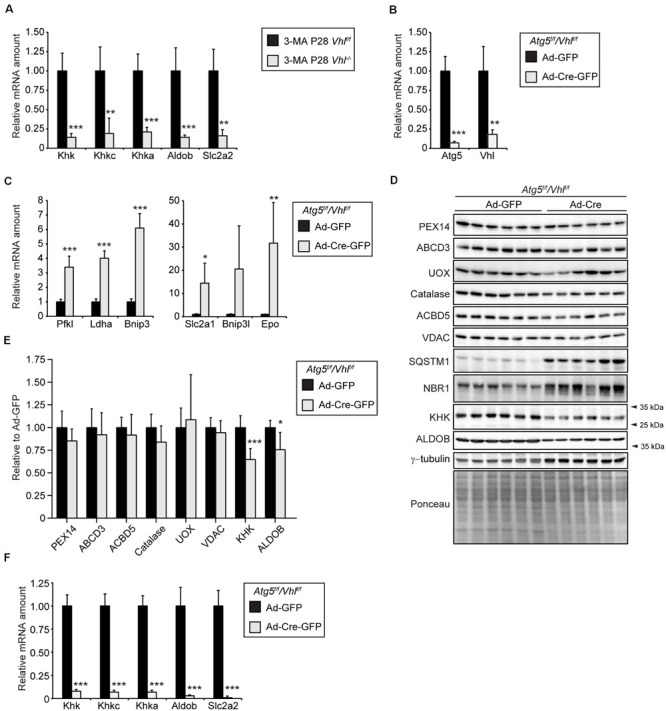
HIF-2α-mediated decrease of the expression of fructolytic genes does not depend on decreased peroxisome abundance. **(A)** Expression of fructolytic genes in livers from vehicle- and 3-MA-treated control and *Vhl^–/–^* mice (*n* = 7 for vehicle-treated control mice; *n* = 3 for 3-MA-treated *Vhl^–/–^* mice. **(B)** 15-week-old *Atg5^*f/f*^/Vhl^*f/f*^* mice were infected with 3 × 10^9^ pfu of Ad-GFP or Ad-Cre-GFP and sacrificed after 6 days (*n* = 6 for Ad-GFP and Ad-Cre-GFP). The efficiency of *Atg5* and *Vhl* deletion was measured by quantifying *Atg5* and *Vhl* mRNA by quantitative RT-PCR. **(C)** Expression of HIF-α target genes. **(D)** Immunoblots of liver lysates. **(E)** Quantification of immunoblots after normalization to ponceau. **(F)** Expression of fructolytic genes. Each value represents the amount of mRNA relative to that in control mice, which was arbitrarily defined as 1. *Cyclophilin* was used as the invariant control. Data are mean ± SD. Statistical analysis was performed using Student’s *t-*test or Student’s *t-*test with Welch’s correction. *p < 0.05; **p < 0.01; ***p < 0.001 vs. control mice.

Next, we wanted to investigate whether the decrease in mRNA levels of fructolytic genes caused by HIF-2α signaling in *Vhl^–/–^* and *Vhl^–/–^*/*Hif1a^–/–^* mice is due to the decrease in peroxisome number caused by HIF-2α-induced pexophagy. We have previously shown that pharmacological inhibition of autophagy with 3-methyladenine (3-MA) restores peroxisome homeostasis in livers of *Vhl^–/–^* mice after 5 days of 3-MA treatment (Walter et al., 2014). Hif target gene expression was not affected by 3-MA treatment. The mRNA levels of *Khk*, *Khkc*, *Khka*, *Aldob*, and *Slc2a2* were decreased to the same extent in 3-MA-treated P28 *Vhl^–/–^* mice (Figure 9A) as in untreated *Vhl^–/–^* mice (Figure 1D).

We used an additional approach to show that the HIF-2α signaling pathway reduces the expression of fructolytic genes independent of peroxisome number. Therefore, we examined the effect of constitutive HIF-α signaling on peroxisome abundance and the expression of fructolytic genes in liver-specific, autophagy-deficient *Atg5^–/–^/Vhl^–/–^* mice. Liver-targeted deletion of *Atg5* and *Vhl* was achieved by systemic administration, via tail vein injection, of an adenovirus co-expressing Cre recombinase and GFP (Ad-Cre-GFP) or Ad-GFP as control to 15-week-old *Atg5^*f/f*^/Vhl^*f/f*^* mice. Mice were sacrificed 6 days after the injection of adenoviruses. Expression analysis by qRT-PCR confirmed the efficient deletion of *Atg5* and *Vhl* from the liver (Figure 9B). The mRNA levels of HIF target genes (*Pfkl*, *Ldha*, *Bnip3*, *Bnip3l*, *Slc2a1*, and *Epo*) were significantly increased in livers after deletion of *Vhl* (Figure 9C). The concomitant deletion of *Atg5* and *Vhl* prevents the loss of peroxisomes by HIF-2α-induced pexophagy. The protein levels of the peroxisomal matrix proteins catalase and urate oxidase (UOX), the peroxisomal membrane proteins PEX14, ABCD3, and ACBD5, and the mitochondrial outer membrane protein VDAC were similar in *Atg5^*f/f*^/Vhl^*f/f*^* and *Atg5^–/–^/Vhl^–/–^* livers (Figures 9D,E). The protein levels of the selective autophagy receptors sequestosome 1 (SQSTM1/p62) and neighbor of BRCA1 gene 1 (NBR1), which are involved in HIF-2α-mediated pexophagy, were significantly increased in *Atg5^–/–^/Vhl^–/–^* livers compared to controls (Figure 9D). The accumulation of NBR1 and SQSTM1 is a confirmation that both macroautophagy and HIF-2α-mediated pexophagy were inhibited in *Atg5^–/–^/Vhl^–/–^* livers. Importantly, the expression of fructolytic genes was strongly decreased in *Atg5^–/–^/Vhl^–/–^* livers (Figure 9F). The protein levels of KHK and ALDOB were significantly reduced in *Atg5^–/–^/Vhl^–/–^* livers (∼ 40% and ∼25% reduction, respectively) (Figures 9D,E), however, the reduction was less prominent than observed in livers of *Vhl^–/–^* and *Vhl^–/–^/Hif1a^–/–^* mice (Figures 1F,G, 2E). A similar observation was made in HIF-2α(mt)-expressing livers of mice (Figure 3B), which were also sacrificed 6 days after adenovirus injections, which indicates a considerable stability of these proteins.

In summary, we conclude that HIF-2α signaling and peroxisome-deficiency are strong negative regulators of the expression of fructolytic genes. Furthermore, the suppression of fructolytic genes by HIF-2α is not dependent on a decrease in the number of peroxisomes by HIF-2α-induced pexophagy.

## Discussion

High fructose consumption and metabolism have been associated with the development of various pathologic conditions. However, still little is known about the general regulation of fructolytic enzymes. The main finding of this study is that in organs with high fructolytic activity total *Khk* and associated *Khkc* and *Khka* expression is negatively regulated by HIF-2α signaling and by lack of peroxisomal metabolism.

KHK, more specifically the high-activity fructose-catabolizing isoform KHK-C, displays the highest expression in the liver followed by the kidney and the intestine (Hayward and Bonthron, 1998; Diggle et al., 2009). The liver and kidney are also characterized by abundant peroxisome levels and are severely affected in patients suffering from peroxisomal disorders (Waterham et al., 2016; Islinger et al., 2018). Peroxisomal disorder patients and Zellweger syndrome mouse models suffer from vast metabolic disarray that includes in particular severe abnormalities in lipid metabolism (Baes et al., 1997; Faust and Hatten, 1997; Maxwell et al., 2003; Baes and Van Veldhoven, 2012; Wanders, 2018). The enzymes of glycolysis and fructolysis, gluconeogenesis, glycogen catabolism, and pentose phosphate pathway are not present in mammalian and *Drosophila* peroxisomes (Wiese et al., 2007; Faust et al., 2012; Gronemeyer et al., 2013). However, a peroxisome-related organelle called glycosome contains the majority of glycolytic enzymes in the protist group Kinetoplastea, which include trypanosomatid parasites of the genera *Trypanosoma* and *Leishmania* (Haanstra et al., 2016). Interestingly, enhanced glycolysis and impaired glycogen synthesis and gluconeogenesis has been shown in 20-week-old liver-specific peroxisome-deficient *Pex5^–/–^* mice (Peeters et al., 2011), and these perturbations were attributed to mitochondrial dysfunction. Reduced levels of glycolytic, glycogen, and pentose phosphate pathway intermediates were found in peroxisome-deficient *Drosophila pex2* and *pex16* mutants, while no dramatic mitochondrial phenotypes were observed in mutant flies (Wangler et al., 2017). We demonstrate a strong decrease in total KHK at the mRNA and protein level, which is also reflected in decreased expression of both the *Khka* and *Khkc* isoforms, in livers from the *Pex2*^–/–^ mouse model for Zellweger syndrome without functional peroxisomes. The repressive effects are enhanced in postnatal and weaned mice (P10 and P36, respectively) compared to newborn (P0) mice. We also observed a decrease of *Aldob*, an enzyme downstream of KHK, and *Slc2a2* at the mRNA level in livers of P0 and P36 *Pex2*^–/–^ mice, whereas their expression was not reduced in P10 *Pex2*^–/–^ livers. Decreased expression levels of *Khk* and *Slc2a2* were also found in a microarray analysis on livers of 20-week-old liver-specific *Pex5^–/–^* mice, while no differences in *Aldob* expression were observed (Peeters et al., 2011). Treating *Pex2^–/–^* mice with bile acids prolonged postnatal survival, alleviated intestinal malabsorption and intrahepatic cholestasis, and reduced production of toxic C_27_-bile acid intermediates (Keane et al., 2007). However, BA therapy exacerbated the degree of hepatic steatosis and worsened the mitochondrial and cellular damage in peroxisome-deficient livers. However, BA feeding had no effect on the expression of fructolytic genes in *Pex2*^–/–^ livers, while *Khk* as well as *Khkc* expression was slightly reduced in P10 control mice compared to untreated mice. With the exception of kidney and intestine, expression of *Khkc* is very low in extrahepatic tissues such as the heart, and the low-activity KHK-A is the predominant isoform under physiologic conditions (Diggle et al., 2009; Chabbert et al., 2019). Interestingly, absence of peroxisomes in *Pex2^–/–^* mice resulted in an upregulation of the *Khka* isoform, also reflected by upregulation of total *Khk*, in extrahepatic tissues (heart, lung, skeletal muscle and spleen) at the transcriptional level. The *Khkc* isoform remained expressed below detection levels and even total KHK protein was not detectable, probably due to the low *Khk* expression levels. This indicates that loss of peroxisomes reduces total KHK expression in tissues with high fructolytic activity but does not cause an isoform switch, neither toward *Khkc* nor *Khka*.

Previously, we demonstrated that hypoxic signaling in liver-specific *Vhl^–/–^* mice triggers selective degradation of peroxisomes specifically via the HIF-2α isoform (Walter et al., 2014). Surprisingly, we also observed a strong repression of KHK and ALDOB by HIF-2α signaling in the liver, while HIF-1α signaling did not affect their expression. Even in 11-month-old *Vhl^–/–^/Epas1^–/–^* mice we only observed minor hepatic *Khk* expression changes despite HIF-1α stabilization over such a long period. Whereas the peroxisome abundance was not yet reduced in livers of P7 *Vhl^–/–^* mice, the expression of HIF-α target genes was highly induced and the mRNA and protein levels of KHK were already strongly reduced. These observations suggested that the suppression of *KHK* expression by HIF-2α is not dependent on a decrease in the number of peroxisomes by HIF-2α-induced pexophagy. Indeed, hepatic mRNA levels of fructolytic genes were also decreased in 3-MA-treated P28 *Vhl^–/–^* and 15-week-old *Atg5^–/–^/Vhl^–/–^* mice, where HIF-2α signaling could not decrease the number of peroxisomes due to the inhibition of autophagy.

HIF-1α and HIF-2α have both overlapping and distinct target genes as well as different cellular expression patterns (Hu et al., 2003; Sowter et al., 2003; Nakazawa et al., 2016). They are differentially regulated in various physiological settings and function in pathophysiological conditions such as cancer and ischemic diseases (Semenza, 2012). They have different roles in tumorigenesis dependent on specific tumor microenvironments (Keith et al., 2011). HIF-2α is a major oncogenic driver in clear cell renal carcinoma (ccRCC) where HIF-1α acts as tumor suppressor (Kondo et al., 2002; Raval et al., 2005; Gordan et al., 2007; Shen et al., 2011). Interestingly, reduced KHK levels were found in human ccRCC tumors compared to normal renal tissue (Hwa et al., 2006; Neely et al., 2016). HIF-2α might be responsible for the KHK downregulation and it would be interesting to understand how fructose metabolism is affected in these tumors. In contrast, tumor-promoting roles have been described for HIF-1α while HIF-2α inhibited tumorigenesis in colon cancer (Imamura et al., 2009). High-fructose corn syrup and elevated fructose metabolism have been shown to enhance intestinal tumor growth in adenomatous polyposis coli (*Apc*) knockout mice, and *Khk* knockout abolished high-fructose corn syrup enhancement of tumor growth and grade (Goncalves et al., 2019). Elevated KHK protein levels have also been detected in human colorectal adenomas compared to normal colon tissue (Uzozie et al., 2014). It is tempting to hypothesize that a tumor-promoting effect of fructose might only be observed in *Epas1*-deficient intestinal tumors.

It remains an open question how HIF-2α represses *Khk* and *Aldob* expression, but several possibilities exist. Studies using chromatin immunoprecipitation of HIF-α subunits coupled to either microarray analyses or next-generation DNA sequencing defined an identical core binding motif (RCGTG) for HIF-1α and HIF-2α, but binding to this motif is highly selective and the pathways targeted by HIF-1α and HIF-2α differ considerably (Mole et al., 2009; Schodel et al., 2011). That means that additional factors (e.g., epigenetic or other transcription factors) are involved in directing HIF-1α and HIF-2α to their target genes. Expression array studies have defined similar numbers of genes that are positively and negatively regulated by HIF-α. However, when promoters of negatively regulated genes in hypoxia-cultured MCF-7 cells were surveyed for HIF-α binding, an excess of binding over that in the promoters of genes that were entirely unresponsive to HIF-α was not observed (Mole et al., 2009). Therefore, it has been hypothesized that for the large majority of genes HIF-α-dependent down-regulation of expression is likely to be due to indirect effects *“in trans*,*”* rather than direct effects of HIF-α on the promoter. Indeed, a number of genes encoding transcriptional repressors have been identified as positively regulated HIF-1α targets. The HIF-1α-regulated gene *DEC1/Stra13*, a member of the *Drosophila hairy/Enhancer of split* transcription repressor family, represses *PPAR*γ*2* expression and inhibits thereby adipogenesis (Yun et al., 2002). In human clear cell renal cell carcinoma, it has been shown that the expression of E-cadherin and HIF-1α was mutually exclusive due to HIF-1α-mediated induction of *TCF3*, *ZFHX1A*, and *ZFHX1B*, which repress *E-cadherin* gene transcription (Krishnamachary et al., 2006). Moreover, it has also been proposed that displacement of more powerful transcriptional activators, or recruitment of corepressors to HIF-α, accounts for down-regulation of gene expression by HIF-α. For example, it has been suggested that the repression of α*-fetoprotein* (*AFP*) and *carbamoyl phosphate synthetase-aspartate carbamoyltransferase-dihydroorotase* (*CAD*) expression by HIF-1α is linked to a competition between HIF-1α and c-Myc (Mazure et al., 2002; Chen et al., 2005). In addition, HIF-1α activation increases expression of *MAX-interacting protein 1* (*MXI1*) and *Forkhead-box protein O3a* (*FOXO3a*) and thereby inhibits mitochondrial biogenesis by reducing MYC activity (Schönenberger and Kovacs, 2015). Recently, the SIN3A histone deacetylase complex and the REST complex have been shown to be involved in hypoxic gene repression, though a direct link to the transcriptional activity of HIF is still missing (Cavadas et al., 2016; Tiana et al., 2018). Regarding HIF-2α, it has also been suggested that post-DNA-binding mechanisms affect transcriptional activity (Hu et al., 2006; Lau et al., 2007).

The adaptation to hypoxic stress via enhanced fructolysis in brain and heart has been shown to prolong survival under anoxic conditions in the naked mole rat *(Heterocephalus glaber)*. Moreover, these animals endured conditions of chronic hypoxia without obvious side effects in which other animal species died (e.g., *Mus musculus*) (Park et al., 2017). Additionally, HIF-1α expression has been described to be elevated in naked mole rats compared to mice under normoxic and hypoxic conditions (Xiao et al., 2017). Nevertheless, further studies are required to understand whether HIF-1α-induced fructose metabolism is involved in the adaptation to chronic hypoxia.

Dietary fructose intake induces SLC2A5 as well as SLC2A2 expression and thereby enhances intestinal fructose absorption, serum levels and uptake (Barone et al., 2009). Furthermore, *Slc2a5* deletion results in reduced expression levels of *Khk* and *Aldob* on mRNA and protein levels in the intestine (Patel et al., 2015a, b). SLC2A5 expression was increased in intestinal tumors of *Apc^–/–^* mice compared to wild-type intestinal epithelial cells while fructose levels in the serum and liver were reduced indicating enhanced uptake by the cancer cells (Goncalves et al., 2019). Moreover, administration of high-fructose corn syrup triggered intestinal tumor growth via increased levels of glycolysis and fatty acid synthesis using glucose and fructose as substrate and these effects were reversed in *Apc^–/–^/Khk^–/–^* mice (Goncalves et al., 2019). Hypoxia has been shown to upregulate *Slc2a5* in adipocytes on mRNA but not on protein levels (Wood et al., 2007). While *Slc2a5* expression remained below detection levels in the liver, we observed a significant downregulation of *Slc2a5* mRNA in kidneys of *Pex2^–/–^* mice. Interestingly, *Slc2a2* is transcriptionally downregulated in P0 and P36 but not in P10 livers of *Pex2^–/–^* mice. Additionally, *Slc2a2* was significantly downregulated only in P28 *Vhl^–/–^* and *Vhl^–/–^/Hif1*α*^–/–^* livers while *Khk* and *Aldob* expression was already strongly reduced in P7 and P14 *Vhl^–/–^ livers*. Taken together, these results indicate that HIF-2α signaling and peroxisome-deficiency reduce fructose metabolism mainly via downregulation of *Khk* which might be followed by reduced expression of fructose transporters.

A study reported that acute myeloid leukemia (AML) cells express high levels of SLC2A5 and consume fructose and use it to maintain viability, especially when glucose is scarce (Chen et al., 2016). Studies have shown that fructose utilization can drive more efficient incorporation of carbohydrates into proteins and nucleic acids and can act as a more efficient fuel for the pentose phosphate cycle, when compared to glucose (Laughlin, 2014). This metabolic feature might create an important biological target since most normal cells including normal monocytes do not use fructose as main metabolic fuel. Progression of AML has been linked to the expansion of hypoxia and HIF-1α signaling in the sub-endosteal bone marrow niche relative to normal bone marrow (Tabe and Konopleva, 2014). Another study showed that SLC2A5 is significantly upregulated in lung adenocarcinoma patients and overexpression of SLC2A5 determines fructose uptake and utilization efficacy and is highly correlated with poor patient survival (Weng et al., 2018). However, it is not known whether enhanced fructose utilization in AML cells and lung adenocarcinoma is enabled by a HIF-1α-mediated switch to *KHKC* expression.

A study showed that myocardial hypoxia triggers fructose metabolism via a HIF-1α-induced and SF3B1-mediated splicing switch from *Khka* to *Khkc* in mice. In accordance with HIF-1α induction, elevated KHK-C and SF3B1 levels were also detected in biopsies from patients suffering from aortic stenosis or hypertrophic cardiomyopathy (Mirtschink et al., 2015). Recently, an isoform switch from *KHKC* to *KHKA* mediated by a c-MYC-mediated upregulation of the splicing factors HNRNPH1/2 has been shown to promote the progression of hepatocellular carcinoma in human cells (Li et al., 2016). The malignant cells thereby reduce their normally high fructose metabolism in favor of alternative metabolic pathways that permit proliferation. Additionally, two studies showed an involvement of A1CF in *Khk* splicing (Lin et al., 2018; Nikolaou et al., 2019). A1CF has been described to be involved in the regulation of several metabolic genes and to be sensitive to metabolic changes, dynamically shuttling between cytosol and nucleus from fed to fasted state (Sowden et al., 2004; Lehmann et al., 2006; Lin et al., 2018; Nikolaou et al., 2019). Nikolaou et al. (2019) described A1CF to be a positive regulator of KHK-C isoform expression while inhibiting KHK-A expression with HNRNPH1/2 displaying opposite effects. Liver-specific *A1cf^–/–^* mice revealed reduced KHK activity and KHK-C protein levels as well as a slight downregulation of peroxisomal genes (*Crot, Pex5, Pex7, Ehhadh*) (Nikolaou et al., 2019). We did not observe a *Khk* splicing switch in response to HIF-2α signaling or in peroxisome-deficient tissues at the transcriptional level, neither in the liver nor in the kidney. Rather, downregulation of total *Khk* expression is reflected by repression of the *Khkc* as well as the *Khka* isoforms in tissues with high fructolytic capacity. In line, SF3B1 and HNRNPH1/2 appear not to be involved in KHK downregulation in our models and minor changes might be explained by secondary effects since these splicing factors have been linked to the regulation of various genes (Bonnal et al., 2012; Huelga et al., 2012). Interestingly, we observed that HIF-2α signaling increased *A1cf* expression, but A1CF protein levels were lower in total liver lysates as well as cytosolic and nuclear fractions from *Vhl^–/–^* livers. In contrast, A1CF levels were increased in nuclear fractions from livers of both untreated and BA-fed peroxisome-deficient *Pex2^–/–^* mice. Although, we cannot explain the discrepancies between *A1cf* mRNA and protein expression, these results support our conclusion that the decrease of hepatic KHK levels via HIF-2α signaling or due to peroxisome-deficiency is independent of splicing, since both *Khka* and *Khkc* were downregulated. However, these splicing factors might be relevant for the regulation of other genes in peroxisome-deficient livers.

PPARα is a major regulator of lipid metabolism and controls energy homeostasis in the liver (Pyper et al., 2010). Activation of PPARα by fibrates has been shown to ameliorate fructose-induced non-alcoholic steatohepatitis (Abd El-Haleim et al., 2016a, b). PPARα is hyperactivated in the liver and extrahepatic tissues of *Pex2^–/–^* mice (Figures 7, 8) due to accumulation of endogenous ligands such as CoA thioesters of very-long chain and branched-chain fatty acids (Hostetler et al., 2005, 2006), that are metabolized in peroxisomes, while ligand-induced activation of PPARα is impaired by HIF-2α signaling (Walter et al., 2014). Since *Khk* expression was strongly decreased in *Pex2^–/–^* livers and kidneys while *Khk* mRNA levels were increased in other extrahepatic tissues, these expression changes most likely occur independently of PPARα activation. Importantly, PPARα was not causally involved in the hepatic alterations of glycolysis, glycogen synthesis, and gluconeogenesis in liver-specific peroxisome-deficient *Pex5^–/–^* mice (Peeters et al., 2011). Treatment of adult C57BL/6J mice with the PPARα agonist WY for 7 days decreased the hepatic expression of *Khk* and *Khkc* by 40 and 65%, respectively, whereas *Aldob* and *Slc2a2* expression was not affected, and protein levels of KHK and ALDOB were slightly decreased. However, the hepatic expression of fructolytic genes was not affected by a 7-day WY treatment of P28 control and *Vhl^–/–^* mice. In addition, KHK and ALDOB protein levels were slightly decreased in WY-treated control mice, whereas no further decrease could be observed in *Vhl^–/–^* livers. Taken together, pharmacological PPARα activation exhibits at most only moderate effects on KHK and ALDOB mRNA and protein levels, which also appear to depend on age.

KHK has been shown to be dispensable for healthy growth and development by using *Khk* as well as *Khka* knockout mouse models (Diggle et al., 2010). High fructose intake triggers *de novo* lipogenesis and promotes features of the metabolic syndrome, including hepatic steatosis and inflammation or elevated levels of serum insulin and triglycerides (Jensen et al., 2018). *Khk*^–/–^ mice are completely protected from the development of these symptoms, specifically via loss of *Khkc* but not *Khka* (Ishimoto et al., 2012, 2013). Additionally, absence or pharmacological inhibition of KHK completely reversed HFI symptoms caused by *Aldob* mutations (Lanaspa et al., 2018). We observed a considerable decrease in KHK expression followed by ALDOB, both enzymes unique for fructose metabolism, on mRNA and protein levels in a Zellweger syndrome mouse model (*Pex2^–/–^* mice). Thus, it would be important to examine whether expression and activity of fructolytic enzymes are also reduced in humans with peroxisomal disorders or whether inhibiting KHK activity would improve clinical prognosis. Fructose has often been used to provide high-calorie nutrition to critically ill patients (Bolder et al., 2009). Adequate caloric intake is crucial for Zellweger patients (Klouwer et al., 2015) and it is important to understand whether peroxisome biogenesis disorder patients can absorb and metabolize fructose or whether fructose is excreted via the urine. In case of impaired fructose metabolism, it might be beneficial to adapt diets of patients and to substitute fructose and sucrose with other nutrients (e.g., glucose and maltose).

**FIGURE 10:**
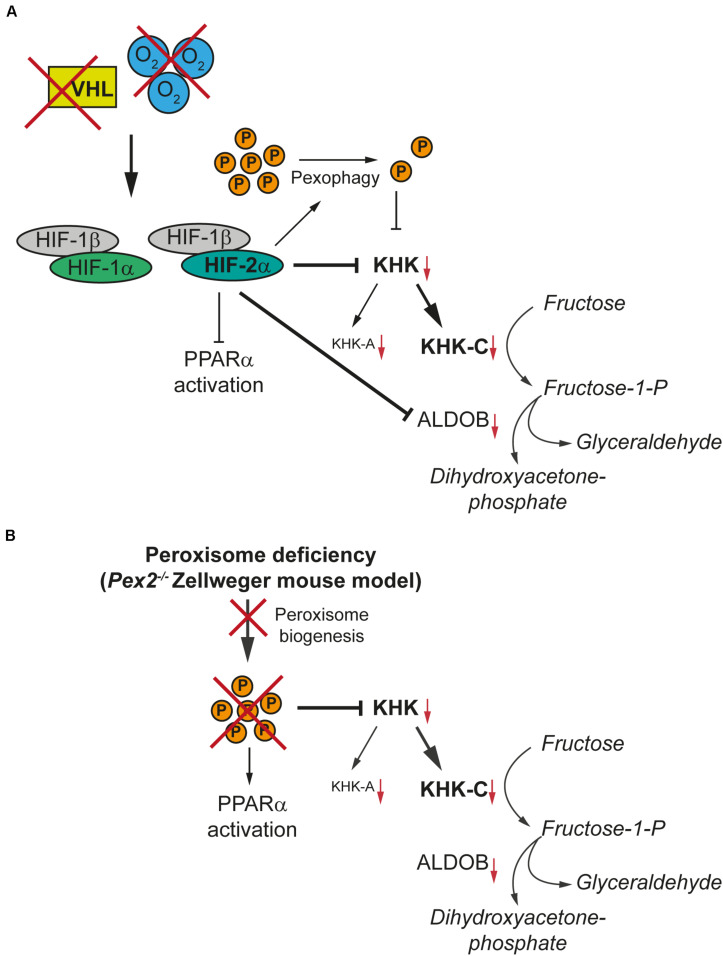
HIF-2α signaling and peroxisome-deficiency reduce KHK expression in tissues with high fructolytic activity. KHK-C is the rate-limiting enzyme of fructolysis and the predominant isoform in tissues with high fructolytic activity, such as the liver and the kidney. **(A)** Hypoxia or loss of pVHL results in the stabilization of HIF-1α and HIF-2α. HIF-2α signaling induces the degradation of peroxisomes (P) via pexophagy and thereby reduces the number of peroxisomes. HIF-2α suppresses total *Khk* and associated *Khkc* and *Khka* expression as well as *Aldob* expression in tissues with high fructose metabolism such as the liver. The suppression of fructolytic genes by HIF-2α is not dependent on a decrease in the number of peroxisomes by HIF-2α-induced pexophagy. **(B)** The lack of functional peroxisomes due to a defect in peroxisome biogenesis also suppresses total *Khk*, *Khkc*, and *Khka* expression in the liver and kidney of the *Pex2^–/–^* Zellweger syndrome mouse model. The absence or a reduced number of peroxisomes and hence decreased peroxisomal metabolic activity leads to the accumulation of very long-chain fatty acids and very long-chain polyunsaturated fatty acids which are potent activators of PPARα. In contrast, HIF-2α signaling represses the ligand-dependent activation of PPARα, which would be a consequence of the decrease in peroxisome number caused by pexophagy. However, PPARα has only a minor effect on fructolysis.

In summary, we identified two negative regulators of KHK expression in major fructolytic organs, namely HIF-2α signaling and peroxisome-deficiency (Figure 10). Both mechanisms suppress total *Khk* and associated *Khkc* and *Khka* expression and are independent of *Khk* alternative splicing and PPARα activation. Hence, this study offers new insights into the general regulation of fructose metabolism as well as an unexpected link between peroxisome function and fructose metabolism.

## Data Availability Statement

The qRT-PCR and western blot raw data generated for this study are available on request to the corresponding author.

## Ethics Statement

The animal study was reviewed and approved by the Veterinary Office of Zurich (Switzerland) and by the Institutional Animal Care and Use Committee of San Diego State University and Columbia University. Written informed consent was obtained from the owners for the participation of their animals in this study.

## Author Contributions

TE and WK conceived and designed the study, performed the experiments, analyzed the data, bred and performed the experiments with Atg5/Vhl mice, and wrote the manuscript. MS, KW, and WK bred and performed the experiments with *Vhl*, *Vhl/Hif1a*, *Vhl/Epas1*, and *Atg7* mice. KC performed the experiments with *Pex2* mice. PF provided the tissues from BA-fed *Pex2* control and knockout mice and contributed to the design of previous studies with *Pex2* mice. WK supervised the project. All authors commented on it and approved the submitted manuscript.

## Conflict of Interest

The authors declare that the research was conducted in the absence of any commercial or financial relationships that could be construed as a potential conflict of interest.

## Abbreviations

A1CF: APOBEC1 complementation factor
ACOX1: acyl-CoA oxidase 1
ALDOB: aldolase B
Atg: autophagy-related protein
BA: bile acid
BNIP3: Bcl-2 and adenovirus E1B 19-kDa-interacting protein 3
BNIP3L/NIX: BNIP3-like
ccRCC: clear cell renal cell carcinoma
CPT1A: carnitine palmitoyltransferase 1A
CRAT: carnitine O-acetyltransferase
CYP4A10: cytochrome P450, family 4, subfamily a, polypeptide 10
EGLN3: egl-9 family hypoxia-inducible factor 3
EHHADH: enoyl-CoA hydratase and 3-hydroxyacyl-CoA dehydrogenase
ENO1: enolase 1
Epas1: endothelial PAS domain protein 1
EPO: erythropoietin
Fructose-1-P: Fructose-1-phosphate
GLUT: glucose transporter
GPI1: glucose-6-phosphate isomerase 1
HFI: hereditary fructose intolerance
HIF: hypoxia-inducible factor
HNRNPH1/2: heterogeneous nuclear ribonucleoprotein H1 and H2
KHK: ketohexokinase
LDHA: lactate dehydrogenase A
NBR1: neighbor of BRCA1 gene
PDGFB: platelet-derived growth factor subunit B
PDK1: pyruvate dehydrogenase kinase 1
Pex: peroxin
PFKL: phosphofructokinase liver-type
PGK1: phosphoglycerate kinase 1
PPAR: peroxisome proliferator-activated receptor
SF3B1: splice factor 3b subunit 1
SLC: solute carrier
SQSTM1/p62: sequestosome 1
TPI1: triosephosphate isomerase 1
UOX: urate oxidase
VDAC: voltage-dependent anion channel
VHL: von Hippel-Lindau
WY: WY-14,643.
