# Supplementary material for: Peroxisome-Deficiency and HIF-2α Signaling Are Negative Regulators of Ketohexokinase Expression
Source: Front Cell Dev Biol. 2020 Jul 8;8:566. doi: 10.3389/fcell.2020.00566 (PMC7360681; doi:10.3389/fcell.2020.00566)
Supplement: Supplementary file 1 [file Data_Sheet_1.pdf]

SUPPLEMENTAL INFORMATION

Peroxisome-deficiency and HIF-2α signaling are negative regulators of ketohexokinase expression

Tanja Eberhart, Miriam J. Schönenberger, Katharina M. Walter, Khanichi N. Charles, Phyllis L. Faust, Werner J. Kovacs

Figure S1, related to Figures 1 and 2

A

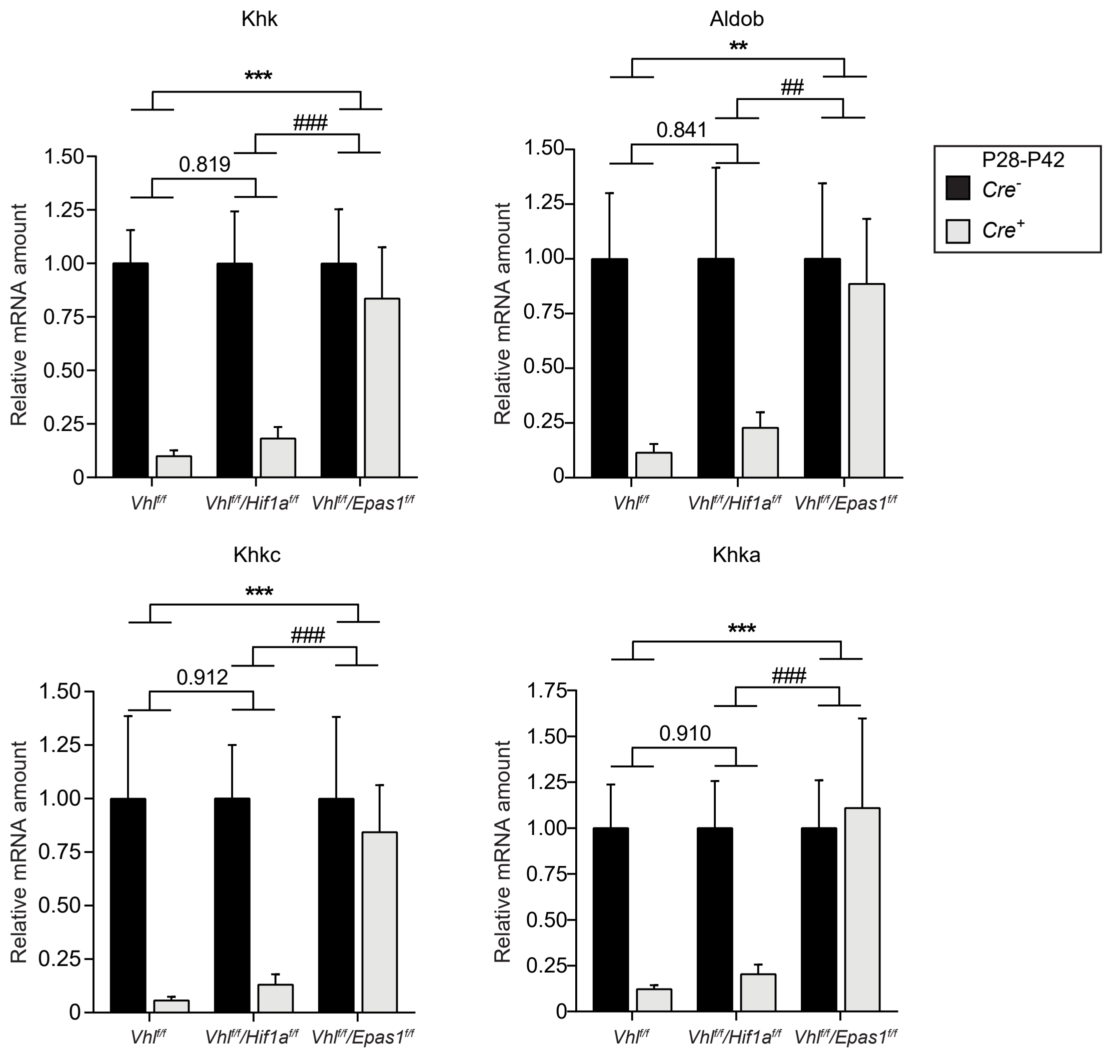

B

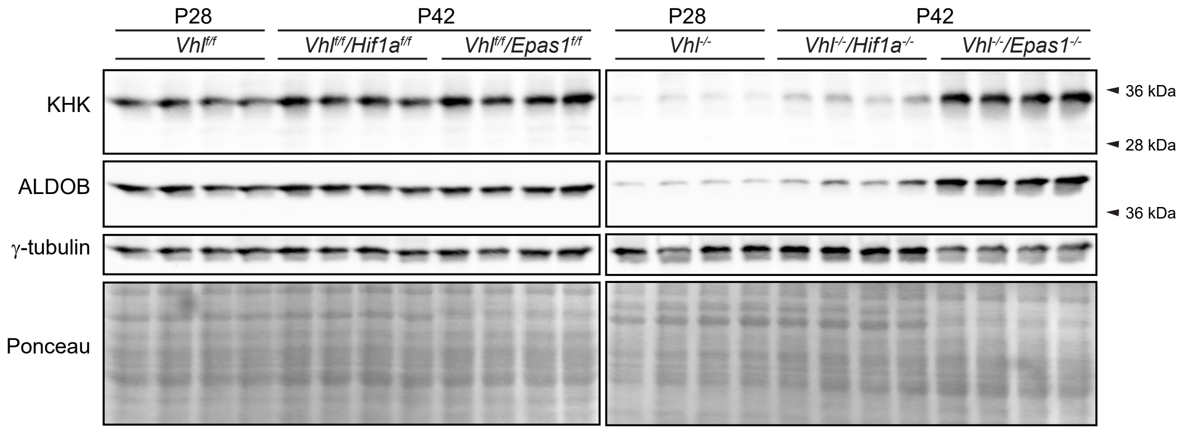

Supplementary Figure S1. Analysis of the fructolytic pathway in *Vhl*$^{-/-}$, *Vhl*$^{-/-}$*/Hif1a*$^{-/-}$ and *Vhl*$^{-/-}$*/Epas1*$^{-/-}$ livers; related to Figures 1 and 2. The fructolytic pathway was analyzed in P28 *Vhl*$^{f/f}$ (control) and liver-specific *Vhl*$^{-/-}$ mice and P28-P42 control (*Cre*-: *Vhl*$^{f/f}$*/Hif1a*$^{f/f}$ and *Vhl*$^{f/f}$*/Epas1*$^{f/f}$) and liver-specific *Vhl*$^{-/-}$*/Hif1a*$^{-/-}$ and *Vhl*$^{-/-}$*/Epas1*$^{-/-}$ mice. (**A**) Statistical evaluation of the hepatic expression changes of fructolytic genes in *Cre*+ mice and their respective *Cre*- control mice between the different mouse strains (i.e., variances between *Vhl*$^{f/f}$ + *Vhl*$^{-/-}$ mice, *Vhl*$^{f/f}$*/Hif1a*$^{f/f}$ + *Vhl*$^{-/-}$*/Hif1a*$^{-/-}$ mice, and *Vhl*$^{f/f}$*/Epas1*$^{f/f}$ + *Vhl*$^{-/-}$*/Epas1*$^{-/-}$ mice). A 2-way ANOVA followed by a Tukey's multiple comparisons test was performed using GraphPad Prism 8.2.0. Each value represents the amount of mRNA in *Vhl*$^{-/-}$, *Vhl*$^{-/-}$*/Hif1a*$^{-/-}$ and *Vhl*$^{-/-}$*/Epas1*$^{-/-}$ mice relative to that in respective control mice, which was arbitrarily defined as 1. *Cyclophilin* was used as the invariant control. Data are mean ± SD (n = 7 for *Vhl*$^{f/f}$ and *Vhl*$^{-/-}$ mice; n = 10 for *Vhl*$^{f/f}$*/Hif1a*$^{f/f}$, *Vhl*$^{-/-}$*/Hif1a*$^{-/-}$, *Vhl*$^{f/f}$*/Epas1*$^{f/f}$, and *Vhl*$^{-/-}$*/Epas1*$^{-/-}$ mice). **, $p < 0.01$; ***, $p < 0.001$; ##, $p < 0.01$; ###, $p < 0.001$. (**B**) Immunoblots of liver lysates from P28-P42 mice with antibodies against total KHK, ALDOB, and γ-tubulin as loading control. Note that total KHK and ALDOB protein levels were not decreased in *Vhl*$^{-/-}$*/Epas1*$^{-/-}$ livers, where only HIF-1α was stabilized and active.

Table S1. Antibodies for western blot analysis

Target	Host	Dilution	Source (Product number, company)
KHK	Mouse	1:500	Sc-377411, Santa Cruz Biotechnology
Aldolase B	Rabbit	1:2000	ab153828, Abcam
ACOX1	Rabbit	0.01 μg/μl	Gift from A. Völkl and D. Fahimi [(1)]
EHHADH	Rabbit	0.01 μg/μl	Gift from A. Völkl and D. Fahimi [(1)]
ABCD3	Mouse	1:1000	SAB4200181, Sigma-Aldrich
ACBD5	Rabbit	1:1000	21080-1-AP, Proteintech
Catalase	Rabbit	1:8000	208910, Calbiochem
PEX14	Rabbit	1:1000	10594-1-AP, Proteintech
β-Actin	Mouse	1:5000	A5316, Sigma-Aldrich
γ-Tubulin	Mouse	1:5000	T6557, Sigma-Aldrich
α-Tubulin	Rabbit	1:5000	ab18251, Abcam
SF3B1	Rabbit	1:1000	ab172634, Abcam
A1CF	Rabbit	1:1000	PA5-60608, Invitrogen
HNRNPH1/2	Rabbit	1:1000	ab154894, Abcam
Lamin B1	Rabbit	1:5000	ab16048, Abcam
NBR1	Mouse	1:500	Ab55474, Abcam
SQSTM1	Guinea pig	1:1000	GP62, Progen
UOX	Rabbit	0.01 μg/μl	Gift from A. Völkl and D. Fahimi [(1)]
VDAC	Rabbit	1:5000	AB10527, Merck Millipore

[(1)]Beier, K., Völkl, A., Hashimoto, T. & Fahimi, H.D. (1988). Selective induction of peroxisomal enzymes by the hypolipidemic drug bezafibrate. Detection of modulations by automatic image analysis in conjunction with immunoelectron microscopy and immunoblotting. *Eur. J. Cell Biol.* 46, 383-393.

Table S2. Quantitative real-time PCR primer.

Gene	Species	Forward primer	Reverse primer
Slc2a1 (Glut1)	*Mus musculus*	5’-CAGTTCGGCTATAACACTGGTG-3’	5’- GCCCCCGACAGAGAAGATG-3’
Pfkl	*Mus musculus*	5’-GGAGGCGAGAACATCAAGCC-3’	5’-GCACTGCCAATAATGGTGCC-3’
Eno1	*Mus musculus*	5’-TGCGTCCACTGGCATCTAC-3’	5’-CAGAGCAGGCGCAATAGTTTTA-3’
Gpi1	*Mus musculus*	5’-TCAAGCTGCGCGAACTTTTTG-3’	5’-GGTTCTTGGAGTAGTCCACCAG-3’
Tpi1	*Mus musculus*	5’-CCAGGAAGTTCTTCGTTGGGG-3’	5’-CAAAGTCGATGTAAGCGGTGG-3’
Ldha	*Mus musculus*	5’-TGTCTCCAGCAAAGACTACTGT-3’	5’-GACTGTACTTGACAATGTTGGGA-3’
Bnip3	*Mus musculus*	5’-TCCTGGGTAGAACTGCACTTC-3’	5’-GCTGGGCATCCAACAGTATTT-3’
Bnip3l	*Mus musculus*	5’-ATGTCTCACTTAGTCGAGCCG-3’	5’-CTCATGCTGTGCATCCAGGA-3’
Pdk1	*Mus musculus*	5’-GGACTTCGGGTCAGTGAATGC-3’	5’-TCCTGAGAAGATTGTCGGGGA-3’
Pgk1	*Mus musculus*	5’-ATGTCGCTTTCCAACAAGCTG-3’	5’-GCTCCATTGTCCAAGCAGAAT-3’
Egln3	*Mus musculus*	5’-AGGCAATGGTGGCTTGCTATC-3’	5’-GCGTCCCAATTCTTATTCAGGT-3’
Epo	*Mus musculus*	5’-CCTCATCTGCGACAGTCGAG-3’	5’-ACAACCCATCGTGACATTTTCT-3’
Khk	*Mus musculus*	5’-AGGTCGATCTGACCCGGTT-3’	5’-TCACGGGGCTTCTCTATCTCC-3’
Khkc	*Mus musculus*	5’-GCGTGGATGTGTCTCAAGTG-3’	5’-GGGTCAGATCGACCTTCTCA-3’
Khka	*Mus musculus*	5’-TTGCCGATTTTGTCCTGGAT-3’	5’-CCTCGGTCTGAAGGACCACAT-3’
Aldob	*Mus musculus*	5’-GAAACCGCCTGCAAAGGATAA-3’	5’-GAGGGTCTCGTGGAAAAGGAT-3’
Slc2a2 (Glut2)	*Mus musculus*	5’-TCAGAAGACAAGATCACCGGA-3’	5’-GCTGGTGTGACTGTAAGTGGG-3’
Pdgfb	*Mus musculus*	5’-CATCCGCTCCTTTGATGATCTT-3’	5’-GTGCTCGGGTCATGTTCAAGT-3’
Pkm2	*Mus musculus*	5’-CGCCTGGACATTGACTCTG-3’	5’-GAAATTCAGCCGAGCCACATT-3’
Slc2a5 (Glut5)	*Mus musculus*	5’-CCAATATGGGTACAACGTAGCTG-3’	5’-GCGTCAAGGTGAAGGACTCAATA-3’
Pex11a	*Mus musculus*	5’-GACGCCTTCATCCGAGTCG-3’	5’-CGGCCTCTTTGTCAGCTTTAGA-3’
Acox1	*Mus musculus*	5’-TCCAGACTTCCAACATGAGGA-3’	5’-CTGGGCGTAGGTGCCAATTA-3’
Ehhadh	*Mus musculus*	5’-ATGGCTGAGTATCTGAGGCTG-3’	5’-GGTCCAAACTAGCTTTCTGGAG-3’

Cpt1a	*Mus musculus*	5’-CTCCGCCTGAGCCATGAAG-3’	5’-CACCAGTGATGATGCCATTCT-3’
Cyp4a10	*Mus musculus*	5’-TTCCCTGATGGACGCTCTTTA-3’	5’-GCAAACCTGGAAGGGTCAAAC-3’
Crat	*Mus musculus*	5’-CAGCCCATCGTGAGTGAGG-3’	5’-CGGACAGCCAGTTCTCCATTT-3’
Sf3b1	*Mus musculus*	5’-GTGGGCCTTGATTCCACAGG-3’	5’-GGCTTCTTCTGACCGAGCAA-3’
A1cf	*Mus musculus*	5’-TGTAGCTGTTGATCCCACTCT-3’	5’-CTGGTGTTTTGGCTCGTGT-3’
Hnrnph1	*Mus musculus*	5’-AAATGGGGCTCAAGGTATTCG-3’	5’-GGACCAGTATGCTTCAACACC-3’
Hnrnph2	*Mus musculus*	5’-GGAGGGGTTCGTGGTGAAG-3’	5’-GAACACCTGATGTGCCATTTTG-3’
18S rRNA	*Mus musculus*	5’-GTTCCGACCATAAACGATGCC-3’	5’-TGGTGGTGCCCTTCCGTCAAT-3’
Ppia	*Mus musculus*	5’-GAGCTGTTTGCAGACAAAGTTC-3’	5’-CCCTGGCACATGAATCCTGG-3’
Atg5	*Mus musculus*	5’-TGTGCTTCGAGATGTGTGGTT-3’	5’-ACCAACGTCAAATAGCTGACTC-3’
Vhl	*Mus musculus*	5’-CATCAGCTACCGAGGTCAT-3’	5’-ACATTGAGGGATGGCACAAAC-3’
